# Tryptophan Side-Chain Oxidase Enzyme Suppresses Hepatocellular Carcinoma Growth through Degradation of Tryptophan

**DOI:** 10.3390/ijms222212428

**Published:** 2021-11-18

**Authors:** Yang Ai, Ben Wang, Shuai Xiao, Sang Luo, Yefu Wang

**Affiliations:** The State Key Laboratory of Virology, College of Life Sciences, Wuhan University, 299 BaYi Road, Wuhan 430065, China; aiyang9311@163.com (Y.A.); 2019202040019@whu.edu.cn (B.W.); xiaoshuai825@hotmail.com (S.X.); 2018102040017@whu.edu.cn (S.L.)

**Keywords:** hepatocellular carcinoma, tryptophan metabolism, enzyme, *de novo* sequencing, anticancer drugs

## Abstract

Tryptophan metabolism plays a role in the occurrence and development of hepatocellular carcinoma cells. By degrading certain amino acids, tumor growth can be limited while maintaining the body’s normal nutritional requirements. Tryptophan side-chain oxidase (TSO) enzyme can degrade tryptophan, and its inhibitory effect on hepatocellular carcinoma cells is worthy of further study. To investigate the degradation effect on tryptophan, TSO was isolated and purified from qq Pseudomonas. The reaction products were identified with high performance liquid chromatography (HPLC) and high-performance liquid chromatography tandem mass spectrometry (HPLC-MS). De novo sequencing provided the complete amino acid sequence of TSO. The results of CCK-8, colony formation, transwell, and qPCR confirmed that TSO had inhibitory effects on the proliferation and migration of HCCLM3 (human hepatocarcinoma cell line) and HepG2 cells. The results of flow cytometry confirmed its apoptotic activity. In animal experiments, we found that the tumor-suppressive effect was better in the oncotherapy group than the intraperitoneal injection group. The results of immunohistochemistry also suggested that TSO could inhibit proliferation and promote apoptosis. In conclusion, a specific enzyme that can degrade tryptophan and inhibit the growth of hepatoma cells was authenticated, and its basic information was obtained by extraction/purification and amino acid sequencing.

## 1. Introduction

Hepatocellular carcinoma (HCC) is one of the most common malignant tumors worldwide, with more than 800,000 new cases and deaths each year [1]. The incidence and mortality rates of HCC are the fourth and third highest, respectively, in China, and the proportion of HCC patients in China is more than 50% [2]. Although the occurrence and development of HCC have been intensively studied in recent years, the precise molecular mechanisms underlying the occurrence of HCC remain unclear. At present, tumor treatments mainly consist of surgery, chemoradiotherapy, immunotherapy, and small molecule compounds [3]. Recent studies have shown that certain amino acids play an important role in the occurrence and development of tumors. These studies have included detection of potential tumor biomarkers [4,5], amino acid derivatives for the treatment of tumors [6,7], elucidation of tumor mechanisms [8,9], and targeted therapy for tumors [10,11].

In 2011, the reprogramming of the energy metabolism of tumor cells included in the top-10 characteristics of tumor cells summarized by Hanahan and Weinberg has become a new focus of tumor research. The energy of rapidly growing tumor cells is mainly derived from glycolysis, which is known as the Warburg effect [12], confirming that amino-acid-based energy metabolism plays an important role in tumor research. The metabolism of some amino acids (i.e., glycine, arginine, and glutamine) provides raw materials for the synthesis of nucleic acids [13,14]. Previous studies on tumor metabolism have shown that tumor metabolic reprogramming can enable tumor cells to maintain selective growth advantages under adverse living conditions [15]. In addition, amino acids also provide the nutritional and biosynthetic bases for immune responses of immune cells and the anabolism of reactive enzymes. Therefore, amino acids can affect tumor growth through their effects on the tumor immune microenvironment [16]. Recent studies have confirmed that a variety of amino acids can directly or indirectly influence the tumor immune microenvironment; hence, modulating the metabolism of tumors via amino acid therapy provides a new direction for tumor-targeted therapies [17].

Tryptophan, first extracted from casein by Hokinst in 1902, is one of the eight essential amino acids that cannot be synthesized in the human body. It is an indispensable component of proteins and plays an important role in maintaining the physiological activities of plants and animals [18]. Tryptophan metabolism is involved in the regulation of immunity, neuronal function, and intestinal homeostasis. In addition to being used for protein synthesis, tryptophan can be broken down by tryptophan hydroxylase (TPH), which is used for the synthesis of 5-hydroxytryptophan (5-HT) and melatonin [19]. Studies have shown that tryptophan metabolism contributes to aging and neurodegeneration by similar mechanisms [20,21]. There are two main tryptophan metabolic pathways: (1) the 5-HT pathway, catalyzed by tryptophan hydroxylase; and (2) the kynurenine pathway. More than 90% of tryptophan is metabolized by the kynurenine pathway [22]. The catabolic pathway of tryptophan in mammals is divided into three steps. Tryptophan is first metabolized to kynurenine and is then metabolized to quinoline, after which it finally undergoes complete oxidative decomposition to produce ATP and CO_2_ [23]. The kynurenine pathway metabolizes various enzymes including indoleamine 2,3-dioxygenase1 (IDO1), indoleamine 2,3-dioxygenase2 (IDO2), and tryptophan-2,3-dioxygenase (TDO2). The enhanced activities of these enzymes can make tryptophan overproduce kynurenine, resulting in tryptophan deficiency and the production of large amounts of kynurenine, thus inhibiting the immune system [24]. Moreover, kynurenine is an endogenous ligand that promotes tumor proliferation; it can bind to and activate aryl hydrocarbon receptor (AHR) to exert biological effects, both of which contribute to the occurrence and development of tumors [25]. AHR is a ligand-activated transcription factor that plays a powerful role in immune cells [26].

As the basis of other forms of metabolism (including those of other amino acids), enzymes have become the focus of metabolomics research. In addition to glycolysis, amino acid metabolism, and lipid metabolism, as well as their participation in the reactions of these metabolic pathways, enzymes have gradually attracted more attention and become key targets for the development of cancer treatment [27]. In 1977, Joseph Roberts discovered and isolated a novel enzyme that oxidizes alpha carbon in the indole ring at site 3. Further studies showed that this enzyme can degrade tryptophan and oxidize the side chain of tryptophan to keto-tryptophan [28]. Roberts et al. [29] described the L-tryptophan degradation enzyme, indole-3-acyl alkane-hydroxylase, and named it as L-tryptophan side-chain oxidase. It was later found that TSO is composed of two kinds of isozymes, namely TSO I and TSO II. TSO I is approximately 60 kDa, whereas TSO II is approximately 44 kDa. The third isolated TSO was isolated by Schmer et al. and was named as TSO III, with a molecular weight of 42 kDa [30].

The development of antitumor drugs targeting tryptophan metabolism has been primarily based on the tumor-promoting functions of IDO1 and TDO to develop small-molecule inhibitors of these enzymes for cancer treatments [31,32,33,34]. However, there are currently no known effective enzymes that can directly degrade tryptophan to inhibit tumors. Although Joseph and his team found that TSO degrades tryptophan, they did not study TSO in depth, investigate its anticancer function at the cellular level, or evaluate its efficacy after tumor-forming experiments.

In the present study, TSO produced by *Pseudomonas* fermentation technology was used as a tumor treatment, and we found that it successfully inhibited the growth of tumor cells and further verified this effect in vivo. In addition, HPLC-MS was used to verify that TSO degraded tryptophan in tumor cells and prevented tryptophan from being metabolized in accordance with the canine urine pathway, thus partially inhibiting the proliferation and growth of tumor cells. Finally, we sequenced the amino acids of TSO, laying a foundation for future investigations of its targets and its development as an anticancer drug.

## 2. Results

### 2.1. TSO Purification, Sequencing and Analysis

#### 2.1.1. Extracted and Purified TSO Degrades Tryptophan

Fermentation of *Pseudomonas* was carried out in a 5 L fermenter. The fermentation conditions of pseudomonas are shown in Table 1. The activity of TSO protein collected under stable condition was the highest. The withdrawal rate was approximately 0.31%. The TSO protein extracted from the bacterial broth collected during the stable period was verified to be active.

**Figure 1:**
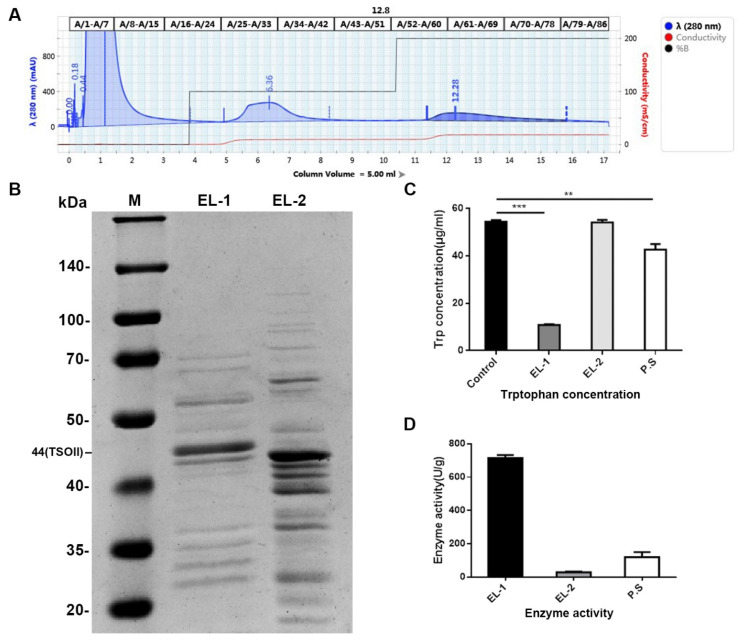
The purified TSO enzyme extracted from Pseudomonas degraded tryptophan. (**A**) Affinity chromatogram of TSO protein. The first peak is the flow-through peak, the second is the 0.05-M sodium acetate elution peak, the third is the 0.2-M sodium acetate elution peak. We collected 11.3–15.7 column volumes of eluent, which was the TSO protein we needed. (**B**) SDS diagrams of proteins in different eluents collected. EL-1 was a strip of 0.2-M eluent and EL-2 was a strip of 0.05-M eluent. In EL-1, the 44-kDa band of protein was TSOII. (**C**) Tryptophan concentration in enzyme activity assay. P.S was the total protein control group. EL-1 had the lowest tryptophan content, indicating the highest protein activity. Data are mean ± SD, *n* = 3; ** *p* < 0.01 and *** *p* < 0.001 vs. Control. (**D**) Enzyme activity of proteins collected from different eluents. The specific activity of EL-1 was significantly higher than that of EL-2.

After the crude enzymatic solution was eluted with 0.05 M of sodium acetate and 0.2 M of sodium acetate on an AKTA protein purifier, pure TSO protein was obtained. Two main peaks with high content were obtained. Elution curves are shown in Figure 1A, which were combined and named as EL-1 and EL-2. The purification efficiency was approximately 78%, as determined by SDS gray-scale analysis (Figure 1B).

Detection of enzymatic activity was carried out on solutions of EL-1, EL-2 and P.S group (total protein). We found that the enzymatic activity of EL-1 on tryptophan was the strongest, while there was little enzymatic activity in the EL-2 eluent (Figure 1C). The specific activities of EL-1 and EL-2 were calculated according to the method of specific enzyme activity calculation. The degradation activity of TSO on tryptophan is shown in Figure 1D. The above results indicate that a large amount of TSO protein existed in 0.2 M of the eluent.

#### 2.1.2. HPLC and HPLC-MS Demonstrate That Tryptophan Is Metabolized by TSO

**Figure 2:**
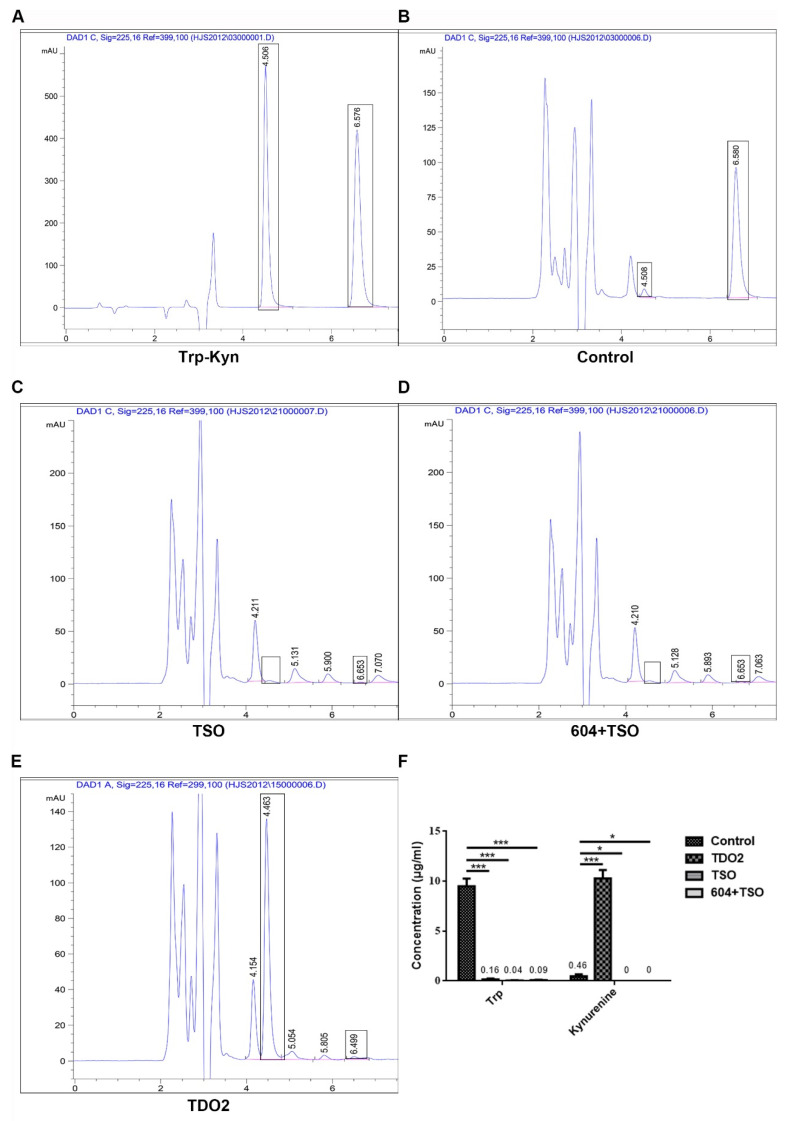

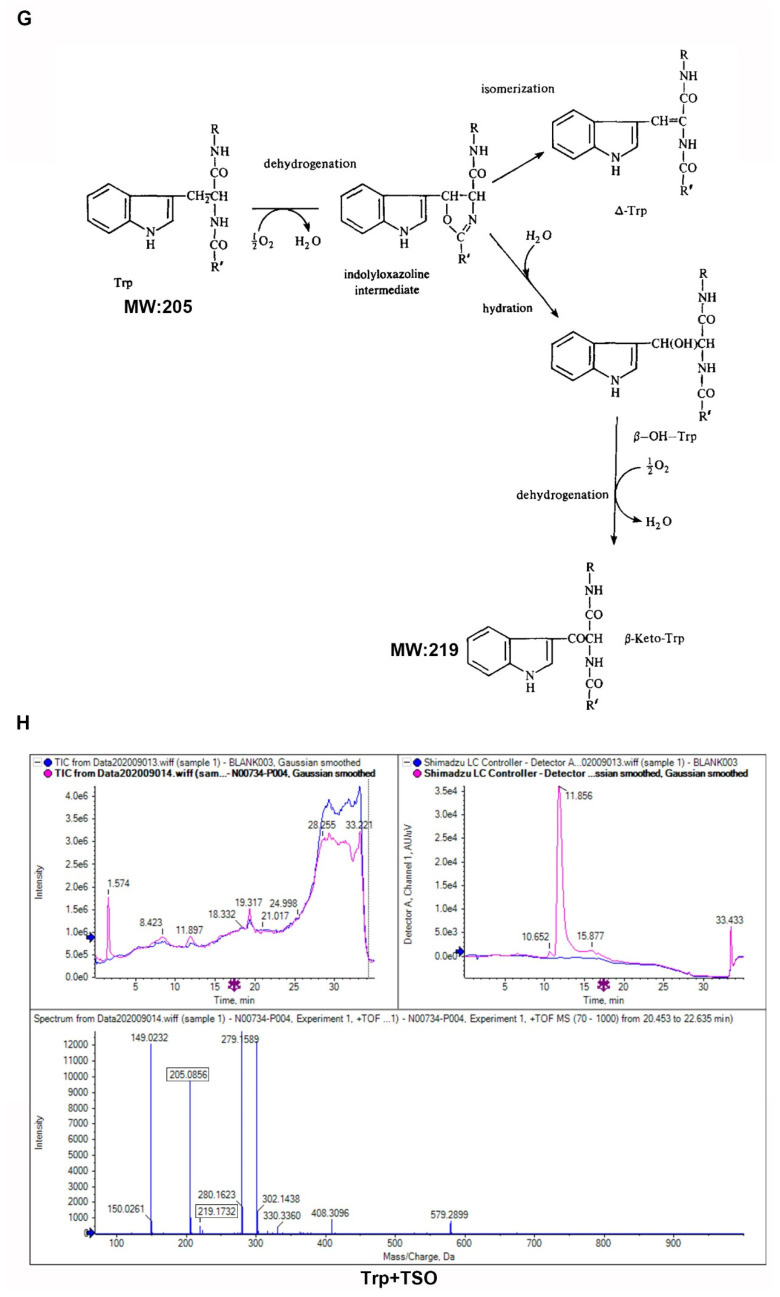
Tryptophan can be completely degraded by TSO. (**A**–**E**) The peak at 4.5 min was kynurenine, and the peak at 6.5 min was tryptophan. 604 was the number of siRNA of TDO2. (**F**) Compared with the normal control group, the contents of tryptophan and kynurenine in the TSO group and the 604+TSO group were almost zero, and almost all the tryptophan was metabolized to kynurenine after transfection with TDO2. Data are mean ± SD, *n* = 3; * *p* < 0.05, and *** *p* < 0.001 vs. Control. (**G**) This reveals the reaction between tryptophan and TSO. Tryptophan with a molecular weight of 204 was eventually metabolized by TSO into a product with a molecular weight of 219. (**H**) HPLC-MS results also confirmed that, in addition to tryptophan (205), the reaction products also contained products with a molecular weight of 219.

As shown in the HPLC results in Figure 2A–F, after transfection with TDO2, all of the tryptophan in the medium was metabolized to kynurenine, while the tryptophan in the medium given TSO alone was degraded but not metabolized to kynurenine, and the medium transfected with the siRNA of TDO2 (this siRNA was synthesized by Sangon biology company and numbered 604, which was verified to have the most obvious inhibitory effect on TDO2 by qPCR in our another manuscript), combined with TSO, also contained no tryptophan and kynurenine.

Based on a previous study [28], the metabolism of tryptophan by TSO is shown in Figure 2G. The results of HPLC-MS (Figure 2H) showed that the product in the reaction of tryptophan and TSO had a molecular weight of 219, which is consistent with the formula shown in Figure 2G. There was no kynurenine (molecular weight of 208) present in the reaction.

#### 2.1.3. Sequence Analysis and Detection of TSO Peptide

**Figure 3:**
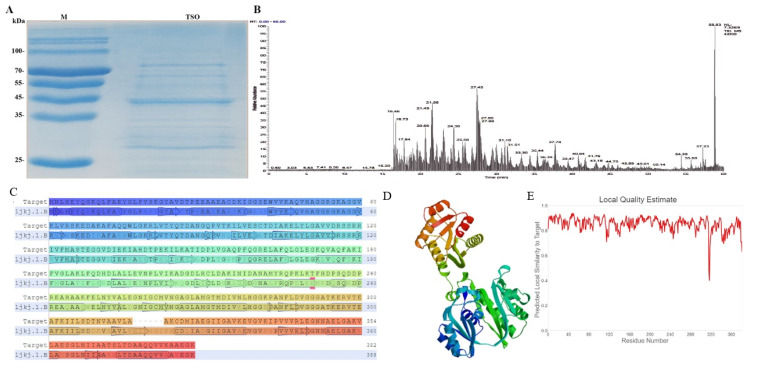
The de novo sequencing analysis of TSO protein and its related properties. (**A**) The band at 44 kDa was purified by SDS-PAGE gelatinization for subsequent mass spectrometry sequencing. (**B**) As can be seen from the total ion-flow diagram, the peak number was large and the peak width was small, indicating that the liquid chromatography separation efficiency was good, and the mass spectrometry data collection was normal. (**C**) Amino acid sequence alignment results on SWISS-MODEL. (**D**) 3D structure predicted by SWISS-MODEL. (**E**) Graphic representation of the accuracy of structural prediction. Ion image of 40 peptides by mass spectrometry sequencing. The X-axis was *m/z*, and the Y-axis was intensity. The pictures are provided in the Supplementary Materials.

The samples were subjected to SDS-PAGE (Figure 3A). Then, colloidal particles with a 44-kD band was cut and treated by protease enzymolysis after affinity chromatography. The processed sample was then analyzed by LC-MS/MS to obtain the raw file of the original MS results. Verification was obtained via Byonic analysis. The raw file generated from the sample data collected by LC-MS/MS was opened using Xcalibur in order to visualize the total ionization chromatogram (Figure 3B). The exact amino acid sequence is shown below:

MNLHEYQGKQLFAEYGLPVSKGYAVDTPEEAAEACDKIGGSEWVVKAQVHAGGRGKAGGVKLVRSKEDAKAFAQQWLGKRLVTYQTDANGQPVTKILVESCTDIAKELYLGAVVDRSSRRIVFMASTEGGVDIEKIAHDTPEKILKATIDPLVGAQPFQGRELAFQLGLEGKQVAQFAKIFVGLAKLFQDHDLALLEVNPLVIKADGDLHCLDAKINIDANAMYRQPKLKTFHDPSQDDPREAHAAKFELNYVALEGNIGCMVNGAGLAMGTMDIVNLHGGKPANFLDVGGGATKERVTEAFKIILSDTNVAAVLAAKCDMIAEGIIGAVKEVGVKIPVVVRLEGNNAELGAKVLA ESGLNIIAATSLTDAAQQVVKAAEGK

The amino acid sequence of TSO was obtained by calculating and stitching the difference of Y-series ions in tandem data, as shown in Supplementary Materials. The MaxQuant software verified that the reliability of the sequence was 100%. The sequences obtained from de novo sequencing were submitted to the NCBI database BLAST for homologous protein retrieval, and it was found that there were highly homologous sequences in the Pseudomonas species (Figure 3C). Although it was highly homologous to the subunit of Succinyl-CoA synthetase reported in the database, this coenzyme A does not have tryptophan degrading activity, and as such they are not the same.

BLAST and HHBlits were used to search the SWISS-MODEL template library for evolution-related structures that matched the target sequence. A total of 96 templates were found. The template with the highest quality was selected for model building. Part of the 3D structure of the TSO protein is shown in the Figure 3D. As shown in Figure 3E, the similarity between amino acids at positions 317–322 and the original TSO sequence decreased sharply, because the sequence did not exist in the original sequence.

The online program ProtParam was used to analyze the physicochemical properties of TSO, which was found to be composed of 382 amino acids. The molecular formula of TSO was found to be C1805H2913N495O545S12, the theoretical isoelectric point (PI) was 5.71, and the estimated half-life was 30 h in mammalian reticulocytes and >20 h in yeast. The instability coefficient was 27.30, which signified that it was a stable protein. The aliphatic index was 98.12, and the grand average of hydropathicity (gravy) was −0.454, showing that was a hydrophilic protein, including 49 negatively charged amino acid residues (Asp + Glu) and 42 positively charged amino acid residues (Arg + Lys).

The TMHMM online prediction showed that the 382 amino acids of TSO were not located in the membrane or the transmembrane, but were instead located in the outer membrane, belonging to membrane-receptor proteins. PSORT II Prediction analysis results showed that 56.5% of TSO was in the cytoplasm, 17.4% was in the nucleus, 17.4% was in mitochondria, 4.3% was in vesicles of the secretory system, and 4.3% was in vacuoles.

### 2.2. TSO Anti-Cancer Funtional Analysis

#### 2.2.1. Inhibitory Effect of TSO in Tumor Cells

As a single drug, TSO showed significant growth inhibition. In our previous study, we also found that when the concentration of TSO was 9 μg/mL (Table 2), it had significant inhibition on positive tumor cells but no obvious effect on normal cells. It was also ensured that endotoxin did not have a cytotoxic effect and the protein concentration was effective.

**Figure 4:**
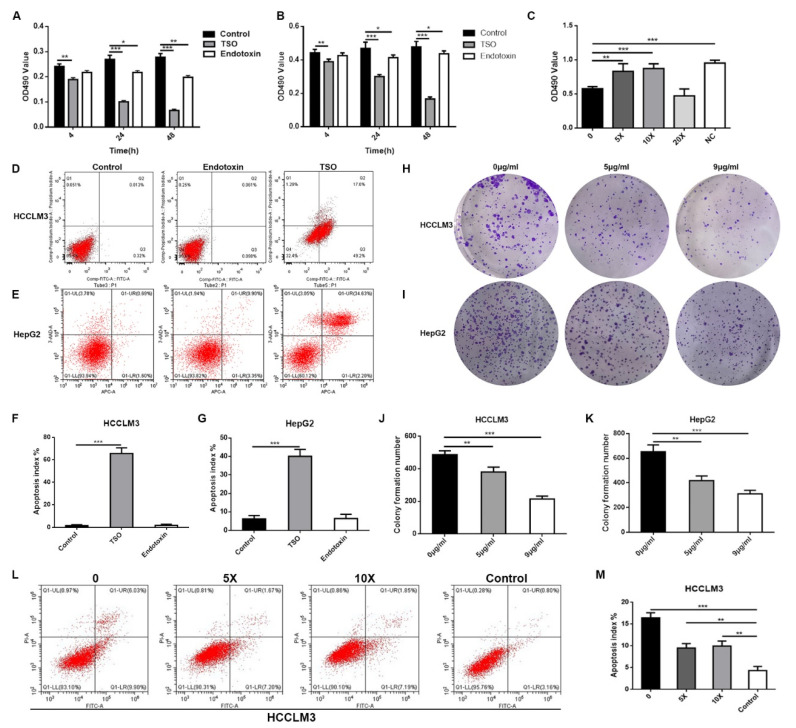
TSO can significantly inhibit the proliferation, migration, and invasion of HCCLM3 and HepG2 cells, and promote apoptosis. (**A**) Effect of TSO on HCCLM3 cell growth determined by CCK-8 assays. Significant inhibitory effects were observed at 4 h. (**B**) Effect of TSO on HepG2 cell growth determined by CCK-8 assays. Endotoxin showed little influence on cell growth. (**C**) DMEM medium containing 10 times as much tryptophan showed the strongest proliferation effect on HCCLM3 cells after TSO administration. Group 0 represents the medium without addition of tryptophan changed after administration. The n× group represented the medium that contained n times as much tryptophan as the original medium changed after administration. The NC group represented the unadministered group. (**D**–**G**) Flow cytometry results of the TSO group, endotoxin group (0.01 EU/mL), and control group. The results showed that TSO could promote the apoptosis of HCCLM3 and HepG2 cells, and that the endotoxin content at this concentration had no effect on cell growth. (**H**–**K**) Colony formation assays in HCCLM3 and HegG2 cells with TSO or control. (**L**,**M**) Flow cytometry results of groups with different tryptophan contents. The culture medium of group 0, 5×, 10×, and control were the same as that of Figure 4C. Data are mean ± SD, *n* = 5; * *p* < 0.05, ** *p* < 0.01 and *** *p* < 0.001 vs. Group 0/Control.

Next, CCK-8 assays were used to measure the anticancer effects of TSO on HCCLM3 cells and HepG2 cells. As shown in Figure 4A, TSO significantly inhibited the proliferation of HCCLM3 cells, and apoptosis was not caused by endotoxins. TSO also had an inhibitory effect on HepG2 cells, while the effect on HepG2 cells was not as obvious as that on HCCLM3 cells (Figure 4B). Flow cytometry results also showed that TSO promoted apoptosis of tumor cells and was not associated with endotoxins (Figure 4D–G). Clone formation assays also confirmed that TSO enhanced the anti-proliferative ability (Figure 4H–K). When the cells were added into the medium containing 5- and 10-time tryptophan, they regained some activity, but not as much as before (Figure 4C,L,M). In contrast, cell growth was inhibited when tryptophan content as high as 20 times was added.

**Figure 5:**
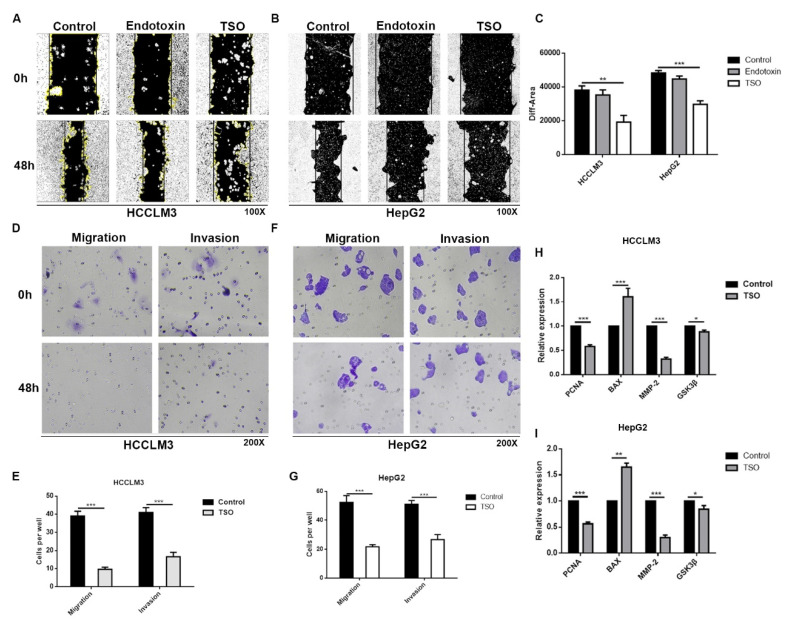
TSO can significantly inhibit the proliferation, migration, and invasion of HCCLM3 and HepG2 cells and promote apoptosis. (**A**–**C**) Wound healing assays showing the inhibition of TSO on the migratory abilities of HCCLM3 cells and HepG2 cells. (**D**–**G**) Transwell assays in HCCLM3 and HepG2 cells with TSO or control. (**H**,**I**) The mRNA level of PCNA, BAX, MMP-2, and GSK-3β in HCCLM3 cells and HepG2 cells after TSO treatment as determined by real-time PCR (SYBR Green). Data are mean ± SD, *n* = 3; * *p* < 0.05, ** *p* < 0.01 and *** *p* < 0.001 vs. Control.

Scratch-wound migration assays also showed that cell migration was significantly inhibited when the cells were subjected to TSO (Figure 5A–C). In summary, TSO significantly inhibited the proliferation of HCCLM3 cells and HepG2 cells and promoted the apoptosis of tumor cells.

Transwell (Figure 5D–G) and experiments also confirmed that TSO significantly inhibited the invasion and migration of HCCLM3 and HepG2 cells.

#### 2.2.2. Effects of TSO on Factors Reflecting Proliferative Changes in Cells

To verify the effects of TSO on cells at the molecular level, we used qPCR to detect cytokines reflective of cell proliferation, apoptosis, and migration. The results are depicted in Figure 5H,I. We observed that compared with those of the normal control group, PCNA and MMP-2 levels in the administration group were significantly decreased, while BAX levels were significantly increased. However, intracellular AKT and GSK3 levels showed little change.

#### 2.2.3. TSO Suppresses the Growth of HCCLM3 Xenograft Tumors

**Figure 6:**
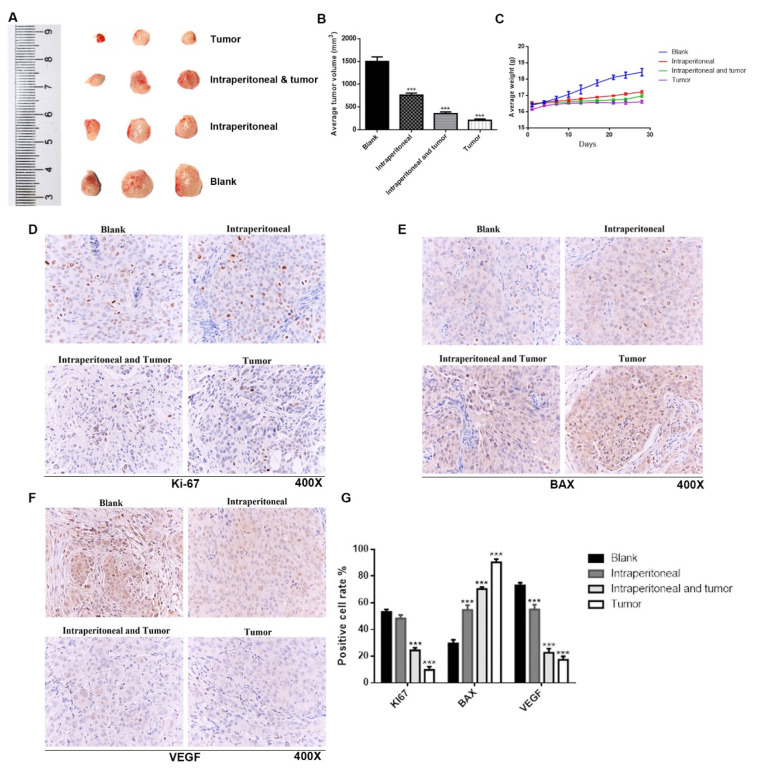
Verification of anticancer effect of TSO in nude mice transplantation experiment. (**A**) BALB/c nude mice were taken for xenotransplantation, and the size was measured by the beside ruler. (**B**) The volume of anatomized subcutaneous xenograft tumors was measured and analyzed by Student’s *t*-test. (**C**) The body weight of nude mice in each group also showed significant difference due to tumor size. (**D**–**G**) Representative Ki-67, BAX and VEGF immunohistochemical staining micrographs of xenograft tumors were performed. Ki-67 and VEGF were significantly reduced after administration. BAX increased significantly after administration. Data are mean ± SD, *n* = 3; *** *p* < 0.001 vs. Blank.

HCCLM3 cells were subcutaneously implanted into the flank of nude mice to study the effects of TSO on tumorigenicity of tumors by measuring the weights and volumes of transplanted tumors in nude mice. The mice were weighed every three days, starting at 7 d after implantations. As shown in Figure 6A–C, the TSO group showed significant inhibition of tumor growth, with the effect increasing in a gradient with the increase of the dose. The tumor inhibition effect was better and more obvious in the oncology administration group than that in the tumor combination group. Immunohistochemical staining results (Figure 6D–G) showed that the positive cell percentage of the proliferation marker Ki-67 and invasion marker VEGF in the tumor administration group were the lowest in the four groups. While the expression level of BAX was the highest in the tumor administration group. These results were consistent with those of our in vitro experiments.

## 3. Discussion

Restricting the intake of certain essential amino acids can cause tumor nutrient deficiency, thus restricting tumor growth or even starving tumors while maintaining the normal nutritional requirements of the body. Therefore, regulating amino acid metabolism in the body may represent a new strategy for anticancer treatments [35,36]. Tryptophan metabolism plays an important role in cancer, which can promote tumor progression by inhibiting anti-tumor immune responses and increasing the malignant properties of cancer cells.

In the present study, we found that TSO can degrade tryptophan. In order to study its degradative activity, we isolated and purified high-purity TSO from Pseudomonas species. Unlike previous methods [29], we selected 0.05 M and 0.2 M sodium acetate buffer for gradient elution, because we found that many TSO proteins were absent in the 0.1 M buffer as described previously. Additionally, our results showed that this gradient elution was more efficient than the 0.1 M buffer, with a purification efficiency of 78%. In order to study the effect of TSO and impurities on the activity, we added the activity of total protein and its degradation ability of tryptophan into the experiment to compare. It was found that under the premise of the same total amount, the higher the purity of TSO was, the stronger its activity was. As shown in Figure 1 of the manuscript, EL-1 had the highest content of TSO and the strongest enzyme activity. Furthermore, we obtained a more concentrated and more active yield of TSO than that found previously using the 0.1 M buffer. We then tested the degradative activity of TSO and verified that it completely degraded tryptophan at 5 μg/mL within 1 h. Therefore, it can be concluded that TSO is an effective component for tryptophan degradation in purified products.

As 95% of tryptophan in human cells is metabolized in accordance with the kynurenine metabolic pathway, HPLC-MS was used to verify that the product produced by tryptophan after TSO metabolism was not kynurenine. In addition, to further verify that TSO had a stronger degradative effect on tryptophan in cells than that of TDO2, HCCLM3 cells were divided into groups and then the contents of tryptophan and kynurenine in each group were detected. We found that tryptophan was completely degraded in the TSO group and in the TDO2 inhibitor combined with TSO group, with almost no kynurenine production. This suggests that TSO was sufficient to metabolize tryptophan in the medium without inhibition of TDO. This result was likely due to the amount of tryptophan in the TSO group being nearly the same as that in the TDO inhibitor group. After verifying that there was no interference of the kynurenine pathway in cells, we confirmed that TSO had a specific and efficient degradative effect on tryptophan. We also found that the combination of a TDO2 inhibitor and TSO completely degraded tryptophan and blocked the metabolism of kynurenine in the body. In subsequent studies, we will investigate the effects of the combination TSO and a TDO2 inhibitor on cancer treatments [37].

In this study, cytological experiments confirmed that TSO had an inhibitory effect on the proliferation, invasion and migration of HCCLM3 and HepG2 cells, which was indirectly supported by QPCR results. It was also proved that sufficient tryptophan could reverse the inhibitory effect of TSO by changing the medium containing different concentrations of tryptophan after administration. In vivo experiments, we found that the tumor growth of the drug administration group was significantly inhibited compared to that of the blank group, as there was a significant difference in tumor size between these groups. The results of immunohistochemistry also confirmed this finding. We also found that the effect of direct tumor administration was better than that of intraperitoneal injection at the same dose, which may be due to the compensatory effect in the body. As studies of ovarian and prostate cancer have shown, cancer-related fibroblasts (CAFs) can resynthesize glutamine and secrete glutamine to support tumor cell growth in a glutamine-restricted environment [38]. However, tryptophan is an essential amino acid that cannot be synthesized by itself. Therefore, it is possible that when tryptophan levels drop to a certain level, the body spontaneously stores tryptophan to provide nutrients for survival. Additionally, through qualitative observations, the mice did not appear anxious or depressed, and continued to eat a normal amount of food. Because tryptophan is not only metabolized by TDO2 to kynurenine, but also a small part of it is metabolized to 5-HT, tryptophan will affect the nervous system and thus affect mood [39]. Hence, this indirectly suggests that a small amount of tryptophan may be stored in the body for maintenance.

There were no tumorigenesis experiments on HepG2 cells because HepG2 cells had been proven to be ineffective in experimental tumor formation. We tried inoculating different sites with different doses of cell suspension, and after 30 days, we did not see any tumor.

Because we used affinity chromatography to purify TSO protein, there were disadvantages of low efficiency and insufficient purity in the subsequent development of drugs. Hence, we also performed de novo sequencing of amino acids for TSO, laying a foundation for the subsequent discovery of its DNA sequence and the efficient production of TSO enzyme by means of genetic engineering. We used the EMBL-EBI software (http://www.ebi.ac.uk/Tools/st/, accessed on 5 January 2021) to predict the probable nucleotide sequence based on the amino acid sequence in Pseudomonas. We then used genetic engineering methods to clone and recombine these sequences to find the nucleotide sequences of proteins with tryptophan degradation activity. In addition, we performed a simple 3D structure prediction of TSO to determine its transmembrane domains and physicochemical properties, which laid a foundation for future studies investigating its targets, receptors, and anticancer function.

By suppressing the proliferation of cancer cells with effective metabolic interventions, it is possible to expand the range of anti-cancer immune responses [40]. For amino acids that are limited, they may be safe to use intermittently for days or weeks, or even for one or two months. Thus, intermittent dietary amino acid restriction may have value and potential as a practical and viable dietary strategy for cancer treatments [41]. This also lays a foundation for the development of colorless amino-acid food for tumor patients.

At present, the regulation of tryptophan metabolism for anticancer treatment is mainly produced by TDO, IDO, and AHR receptor inhibitors [19,42], as well as 5-HT signaling pathways [42]. TSO can directly degrade tryptophan and competitively inhibit TDO2, such that tryptophan is not metabolized in accordance with the kynurenine pathway, thus inhibiting tumor cells. This provides a novel insight into development of anti-tumor drugs and methods. In the future, we will compare the anti-cancer effects of individual or combined administration of a TDO2 inhibitor and TSO.

## 4. Materials and Methods

### 4.1. Cell Cultures, siRNAs, and Transfections

HCCLM3 cells and HepG2 cells were purchased from the National Typical Culture Preservation Center (preservation center of Wuhan University, Wuhan, China). All cell lines were confirmed to be free of mycoplasma contamination, as determined by PCR and culture methods. The species origin of each cell line was confirmed via PCR. The identity of each cell line was authenticated via short tandem repeat (STR) profiling. These cell lines were cultured in DMEM (Hyclone, Logan, UT, USA) containing 10% fetal bovine serum (FBS) (AusgeneX, Queensland, Australia), 100 units/mL penicillin, and 100 μg/mL streptomycin (Hyclone, USA) at 37 °C with 5% CO_2_ in a humidified atmosphere. The medium was replaced every 2–3 d, and the cells were subcultured when their cell fusion rate reached 80–90%. DNA transfection and RNA interference were performed via Lipofectamine 3000 (Thermo Fisher Scientific, Dover, DE, USA), according to the manufacturer’s instructions. The siRNA sense sequence for TDO2 was 5′-CGUGAUAACUUCAAAGGAGAATT-3′ and was synthesized by Sangon Biotech (Shanghai, China). The PCR product of TDO2 was cloned into pEGFP-C1. The complete plasmid was verified by sequencing.

### 4.2. Antibodies and Reagents

The following commercially available antibodies were used: anti-BAX (Servicebio, cat: GB11007-1, 1:50 dilution, Wuhan, China), anti-Ki67 (Abcam, cat: Ab16667, 1:200 dilution, Cambridge, MA, USA), anti-VEGF (Servicebio, cat: GB11007-1, 1:100 dilution, Wuhan, China).

The following commercially available reagents were also used: SDS sample buffer (Sinopharm Group Chemical Reagent Co., Ltd., Beijing, China, cat: 30166428), 30% acrylamide (Biosharp, cat: BL513b, Hefei, China), Trise-Base (Biofavor biotech, cat: 1115GR500, Wuhan, China), Glycine (Biofavor biotech, cat: 1275GR500, Wuhan, China), methyl alcohol, NaCl, KCl, Na_2_HPO_4_.12H_2_O, KH_2_PO_4_, acetic acid, Tween-20 (Sinopharm Group Chemical Reagent Co., Ltd., Beijing, China), protein markers (Thermo Fisher Scientific, Dover, DE, USA).

### 4.3. Aminoagarose Affinity Chromatography Columns

In the first step, 4 g of indoleacrylic acid, 12 g of carbonized dimethylamine, and 4 g of N- hydroxysuccinimide were dissolved in 100 mL of anhydrous dimethylamide. The mixture was placed on a shaker for 24–48 h to produce a pale-yellow ester substance. In the second step, 5 mL of amino agarose filler was added with the same amount of phosphate buffer (0.1 M, pH = 8.5). Then, esters obtained in the first step were added to the balanced amino agarose at a ratio of 1:20, and the reaction was oscillated at room temperature for 1 h. Finally, in the third step, the coupled packing was packed into an empty chromatography column and cleaned with a 0.1 M phosphate solution in a 30× column volume (pH = 6.9). Then, the lower outlet of the column was opened to drain the coupling mixture. The amino agarose affinity chromatography column conjugated with 3-indoleacrylic acid was stored at 4 °C with 0.02% sodium azide.

### 4.4. Thallus Fragmentation Lysates

For thallus fragmentation lysates, 2 mM of EDTA, 100 mM of NaCl, and 0.5% TritonX-100 were dissolved in 50 mM of Tris-HCL (pH = 8.5–9.0). Then, 100 μg/mL of lysozyme and 1 μg/mL of PMSF were added to the lysis buffer before use.

### 4.5. Preparation of Pseudomonas Fermentation Broth

The frozen Pseudomonas strain (ATCC 29574) was inoculated into a sterilized nutrient broth medium and cultured at 30 °C, at 150 rpm for 24 h. Then, the inoculated bacterial solution was added to sterilized 2% BBL liquid medium at a ratio of 1:20, and the mixture was cultured at 200 rpm for 36 h at 30 °C. After the bacteria completed logarithmic growth and reached the stable growth stage (the bacterial content was approximately 2 × 10^10^ cells/mL), the bacteria were centrifuged at 8000 rpm for 1 h at 4 °C, and the storage temperature of the bacterial precipitation obtained by precipitation fermentation was −80 °C. The fermentation conditions of the fermenter were also explored.

### 4.6. Bacteria Lysates

First, 2 g of wet heavy bacteria was taken, and 10 mL of bacteria lysate was added. On an ice bath, the bacteria were lysed by ultrasonication. Ultrasonic crushing conditions were as follows: 140 W, 10 s, an intermittent 10 s, and the working time was 30–40 min. After ultrasonication, the bacterial suspension became clear and transparent, indicating that the bacteria were sufficiently lysed. Then, the liquid after ultrasonic crushing was centrifuged at and 10,000 rpm for 30 min at 4 °C. The supernatant was then collected. Then, the concentration of the extracted protein mixture was detected by ultra-fine ultraviolet spectrophotometry.

### 4.7. Desalination

The crude enzyme liquid was placed into an Amicon Ultra centrifugal filter (the ultrafiltration centrifugal filter underwent four washes with ultra-pure water and was pre-cooled before use). The ultrafiltration centrifuge filter was placed into a centrifuge with the membrane panel facing upward, and was centrifuged at 4000 rpm for 40 min at 4 °C. After centrifugation, the lower layer was drained and then acetate buffer (0.002 M, pH = 5.5) was added to the upper tube to fill the 12 mL calibration line. This step was repeated 2–3 times.

### 4.8. Affinity Chromatography

The enzymatic solution obtained in subsection above was purified by AKTA protein purifier (NGC Chromatography System, Bio-Rad, California, USA). The acetate buffer was balanced with a 30× column volume (0.05 M, pH = 5.5) until the effluent A280 was less than 50 mAU. After equilibrium, the enzymatic solution in the sample ring was added to the affinity chromatography column with 5 mL of acetate buffer (0.2 M pH = 5.5). The loading volume was 1 mL, and the flow rate was 2 mL/min. Then, the eluent was eluted with a 20× column volume of acetic acid buffer (0.05 M, pH = 5.5) and A280 (0.2 M, pH = 5.5) less than 50 mAU, after which the eluent of each gradient concentration of the buffer was collected. Finally, the column was cleaned with a 5× column volume of acetate buffer (0.2 M, pH = 5.5) and ultra-pure water.

### 4.9. Concentration, Endotoxin Removal, and Detection of Enzymatic Activity

The TSO enzymatic solution obtained by washing different concentrations of acetate buffer solution was added into different Amicon ultra centrifugal filters. According to the final required concentration, the TSO enzymatic solution was obtained by centrifugation at 4000 rpm for 30–40 min.

An endotoxin removal kit (ToxinEraser Endotoxin Removal Kit, Genscript, Beijing, China) was used to remove endotoxins from the TSO enzymatic solution. Then, an endotoxin assay kit (ToxinSensor Chromogenic LAL Endotoxin Assay Kit, Genscript, China) was used to measure endotoxin levels in the TSO enzymatic solution to ensure that this solution would not lead to cellular and/or organismal death.

Next, the national standard method was used to detect the content of tryptophan in the reaction solution of TSO and tryptophan to calculate and analyze the enzymatic activity of the TSO enzymatic solution to degrade tryptophan. After the reaction of tryptophan and TSO, the chromogenic substrate was added to detect the absorption value at 590 nm, and the corresponding tryptophan concentration was calculated according to the regression equation.

### 4.10. HPLC and HPLC-MS Detection of Tryptophan and Kynurenine Levels

HPLC-MS was used to detect the metabolites of tryptophan standard substance metabolized by TSO to confirm that TSO metabolized tryptophan and did not activate the kynurenine-metabolizing pathway.

HPLC was performed using an HPLC-MS instrument (Shimadzu, Japan, and ABSciex) for detection and quantitation of tryptophan and kynurenine. The mass scan mode was set to positive and negative ions, and the range was set to 70–1000 *m/z*. This mass spectrometer method was used for the detection of samples eluted from the Shimadzu LC-20 HPLC system. Separation of tryptophan and kynurenine was carried out on an Agilent XDB-C18 column. Standards and samples were eluted using methyl alcohol and ultrapure water (0.1% formic acid). The injection volume was set to 5 μL, with a solvent flow rate of 0.2 mL/min. The column temperature was set to 40 °C. The IDA mode was used for data acquisition.

The contents of tryptophan and kynurenine in cells were determined by HPLC. HCCLM3 cells were divided into the following four groups, with 1 × 10^6^ cells/well that were inoculated into six-well plates: control group, TDO2 group, TSO group, and siRNA combined with TSO group. Then, 2 mL of serum-free medium was added to each well. The culture medium was harvested after 48 h of incubation and was then centrifuged and frozen until HPLC analysis. Standards of known concentrations of kynurenine and tryptophan were used for standardization of assays. Kynurenine and tryptophan reference standards were obtained from Solarbio Life Sciences (Beijing, China). Chromatographic separation conditions are described above.

### 4.11. LC-MS/MS Sequencing

The crude enzymatic solution after affinity chromatography was further purified by SDS-PAGE and the target protein bands (44 kDa) were separated. Trypsin, chymotrypsin, and pepsin were used to enzymolyze the target band.

The enzymatic peptides were analyzed by capillary HPLC (Ultimate 3000, Thermo Fisher Scientific, USA) and electrospray-combined ion-trap Orbitrap mass spectrometry (Q Exactive Hybrid Quadrupole-Orbitrap Mass Spectrometer, Thermo Fisher Scientific, USA). The separation was carried out with prefabricated columns (300 μm i.d. × 5 mm, packed with Acclaim PepMap RPLC C18, 5 μm, 100 Å) and analytical columns (150 μm i.d. × 150 mm, packed with Acclaim PepMap RPLC C18, 1.9 μm, 100 Å). The samples were eluted with mobile phase A (0.1% formic acid and 2% ACN) and phase B (0.1% formic acid and 80% ACN). The solvent flow rate was set to 600 nl/min, and the analysis time of each component was 60 min.

First-order MS parameters were as follows: resolution, 70,000; AGC target, 3,000,000; maximum IT, 40 ms; and scan range, 300–1400 *m/z*. Secondary MS parameters were as follows: resolution, 75,000; AGC target, 100,000; maximum IT, 60 ms; Top N, 20; and NCE/stepped NCE, 27.

The raw MS files were analyzed and searched against a protein database based on the species of the samples using Byonic. The parameters were set as follows. The protein modifications were carbamido methylation (C) (fixed), oxidation (M) (variable), and acetylation (Protein N-term, variable). The enzymatic specificity was set to trypsin. The maximum missed cleavages were set to 3. The precursor ion-mass tolerance was set to 20 ppm, and the MS/MS tolerance was set to 0.02 Da. Only high-confidence identified peptides were chosen for downstream protein-identification analysis. The amino acid sequences were manually spliced and verified by the MaxQuant software.

The amino acid sequences obtained by splicing analysis were performed via BLAST on NCBI (http://www.ncbi.nlm.nih.gov/BLAST, accessed on 4 January 2021), after which the 3D structure was predicted on SWISS-MODEL (http://swissmodel.expasy.org, accessed on 4 January 2021). The ProtParam of ExPASy (http://web.expasy.Org/ProtParam/, accessed on 4 January 2021) was used to predict and analyze physical parameters, such as molecular-weight isoelectric points of proteins. TMHMM software (http://www.cbs.dtu.dk/services/TMHMM-2.0/, accessed on 5 January 2021) was used to predict the transmembrane regions of proteins. Finally, the PSORT II on-line Prediction tool (https://psort.hgc.jp/cgi-bin/runpsort.pl, accessed on 5 January 2021) was used to predict the distribution of TSO protein in cells.

### 4.12. CCK-8 Assays

First, 9 μg/mL of TSO enzyme and 0.01 unit/mL of endotoxin standard were added to 10% DMEM medium as the administration treatment. This dose has been tested for drug endotoxin toxicity (i.e., at this dose, the endotoxin level is below 0.01 units/mL).

HCCLM3 and HepG2 cells were seeded in 96-well plates (2000 cells/well), with 100 μL of medium per well. After 4, 24, and 48 h of incubation, the medium was discarded, and 100 μL of PBS (phosphate buffer saline) and 10 μL of CCK-8 reagent (Beyotime) were added to each well. The cells were then incubated for an additional 1 h. Absorbance values at 450 nm were recorded to assess cell proliferation.

In order to verify the reversal effect of tryptophan on TSO, we studied the changes in cell proliferation and apoptosis under different concentrations of tryptophan. We designed the medium to be replaced with different concentrations of tryptophan (5, 10, and 20 times) 48 h after administration to explore which concentration was most suitable for cell proliferation.

### 4.13. Wound Healing Assays

Exponentially growing cells were inoculated into six-well plates with 5 × 10^5^ cells per well and were divided into the drug treatment group and control group. Linear wounds were scratched with a 0.2-mL pipette tip. The dead cells were washed with PBS. Then, 1 mL of DMEM medium containing 9 μg/mL TSO was added, and drug-free medium was added to the control well. Images were acquired at 0 and 48 h after the cells were wounded. ImageJ (V1.8.0) software was used to measure the uncovered area of cells at each time point.

### 4.14. Detection of Apoptotic Rates via Annexin V-FITC/PI Double Staining

First, for supernatant collection, the culture medium was collected into a flow tube (with a small number of suspended cells). Next, the cells in the six-well plate were washed once with PBS, and 1 mL of 0.25% trypsin was added to digest the cells. When the cells became round and some of the cells were suspended, culture medium was added to stop the digestion. Then, we gently mixed the cells to suspend them. They were collected into a flow tube, centrifuged at 1500 rpm for 5 min, and the supernatant was then discarded. Thereafter, 3 mL of 4 °C pre-cooled PBS was added to resuspend each pellet, which was then centrifuged at 1500 rpm for 5 min. The supernatant was then discarded. The precipitate was resuspended with 300 mL of binding buffer. For fluorescent labeling, 5 Annexin v-fitc and propidium iodide was added and mixed. At room temperature, the reaction was conducted for 5–15 min in the dark. Next, at 1 h after the previous step, Annexin v-FITC green fluorescence was detected by the FITC channel (FL1) and PI red fluorescence was detected by the PI channel (FL2). The parameters for flow cytometry were as follows: excitation wavelength = 488 nm and emission wavelength = 530 nm.

### 4.15. Transwell Invasion Assays

Transwell invasion assays were used to detect the invasive and migratory abilities of cells. First, 3 × 10^5^ cells were inoculated into a precoated supraventral Matrigel (BD Biosciences, NewYork, USA) and DMEM containing 10% fetal bovine serum was added to the underlying Matrigel. After incubation for 24 h, uninvaded cells were removed with cotton swabs, and the invaded cells were fixed with methanol and stained with crystal violet. We then performed microscopy to count the number of invading cells.

### 4.16. Colony Formation Assays

After digestion and counting of logarithmic HCCLM3 and HepG2 cells, 200 cells/well were inoculated into 6-well plates. After 12 h, the medium was changed and TSO treatment at a final concentration of either 5 or 9 μg/mL was added for 48 h. The normal control group was assigned to three wells per group. The cells were placed in an incubator at 37 °C and 5% CO_2_ for two weeks, and the medium was changed every 4 d until visible cell colonies appeared. After formaldehyde was used for fixation, the crystal violet was used for staining and five fields were randomly selected for observation under an inverted microscope. The average cell mass number (greater than 50) was used as the colony count. The experiment was repeated three times.

### 4.17. RNA Extraction and qRT-PCR

Total RNA from cultured cells was extracted using a Takara MiniBEST Universal RNA Extraction kit (Takara Scientific, Inc., Shiga, Japan) and was quantified using a Nanodrop 2000 (Thermo Fisher Scientific, Inc.). cDNA (Complementary DNA) was synthesized from RNA using PrimeScript RT Reagent (Takara Scientific, Inc., Shiga, Japan). Realtime-PCR was performed via an ABI 7500 system (Thermo Fisher Scientific, Dover, DE, USA) using SYBR Premix Ex Taq II (Takara). The primers used were as follows: PCNA forward, 5′-TAATTTCCTGTGCAAAAGACGG-3′ and reverse, 5′-AAGAAGTTCAGGTACCTCAGTG-3′; MMP-2 forward, 5′-ATTGTATTTGATGGCATC-GCTC-3′, and reverse, 5′-ATTCATTCCCTGCAAAGAACAC-3′; GSK-3β forward, 5′-AGGAGAACCCAATGTTTCGTAT-3′ and reverse, 5′-ATCCCCTGGAAATATTGGTTG-T-3′; BAX forward, 5′-CGAACTGGACAGTAACATGG-AG-3′ and reverse 5′-CAGTTTG-CTGGCAAAGTAGAAA-3′; AKT forward, 5′-TGACCATG-AACGAGTTTGAGTA and reverse, 5′-GAGGATCTTCATGGCGTAGTAG-3’; β-actin forw-ard, 5′-TCAAGATCAT-TGCTCCTCCTG-3′, and reverse, 5′-CTGCTTGCTGATCCACATC-TG-3′. The procedures were as follows: one cycle of 95 °C for 30 s, followed by 40 cycles of 95 °C for 5 s, and 60 °C for 34 s.

### 4.18. Tumor Xenograft Assays

A mouse xenograft model was established in five-week-old BALB/c-nu mice (Model Animal Research Institute of the Chinese Academy of Medical Sciences, Beijing, China) in accordance with our institutional guidelines. In brief, 5 × 10^6^ HCCLM3 cells suspended in 100 μL of PBS were injected subcutaneously into the left dorsal flanks of mice. When the tumor size reached approximately 50 mm^3^, 12 mice were equally divided into the following four groups: group A as the blank control, group B as the drug administration group by intraperitoneal injection, group C as the intraperitoneal injection group combined with tumor upper administration, and group D as the tumor administration. Each tumor in group B or D was injected with 50 μL of solution containing 2 mg/mL of TSO. The solution used for intraperitoneal injection and tumor injection in group C contained 50 μL of 1 mg/mL of TSO. We administered the TSO enzyme twice per week for 4 weeks. During treatments, the weight was measured every 3 d. The mice were monitored daily to determine whether they were free to eat, drink, and be active as usual, and whether their body shapes had changed. The behavior and food intake of mice were observed daily. On day 28, the mice were sacrificed, and the tumors were dissected, weighed, and imaged. The tumor volume (V) was calculated using the following equation: V = 0.5 × L × W^2^. The tumor tissues were fixed in 4% paraformaldehyde for further studies. Dewaxing and hydration were performed successively, and then the morphological and structural changes of tumor tissues were observed via microscopy.

### 4.19. Immunohistochemistry

The tumor tissues of the mice were fixed, embedded in paraffin, and sliced into consecutive tissue sections. Next, 5 μm-thick tissue sections were deparaffinized, dehydrated, and heated in citrate buffer (pH = 6.0) for 15 min at 95 °C. To block nonspecific binding of the first antibody, we added 1% bovine serum onto the slides for 20 min at room temperature. Paraffin sections of tumor tissues were taken. BAX (reflecting apoptosis), KI-67 (reflecting proliferation) and VEGF (reflecting invasion) were detected by immunohistochemistry according to the instructions of the SP kit. Finally, the sections were visualized with a DAB kit (ZSGB-bio, Beijing, China) and were counterstained with hematoxylin (Beyotime, Jiangsu, China).

### 4.20. Statistical Analysis

SPSS Statistics 25.0 (Armonk, NY, USA) was used for all data analysis. The data are expressed as the mean ± standard deviation (SD). A *p* value < 0.05 was considered to be statistically significant. Student’s *t*-test was used to evaluate the differences in groups.

## 5. Conclusions

In conclusion, we found that TSO extracted and purified from *Pseudomonas* had a degradative effect on tryptophan and affected proliferation and migration of tumor cells in vitro and in vivo. Collectively, our findings may contribute to the development of anti-tumor drugs. We also hope that our study will be able to combine TSO with other enzyme inhibitors in the future so as to jointly play a stronger anti-cancer effect.

## Figures and Tables

**Table 1:** Fermentation Conditions of Pseudomonas.

Tem (°C)	ph	DO	V (rpm)	Time	State
30	6.26	98.9%	201	0	start
34.3	5.93	28.8%	215	2 h19 m	logarithmic
34.9	4.88	31.1%	201	4 h34 m	logarithmic end
34.5	6.22	66.6%	201	6 h31 m	stable
35.9	7.35	103.3%	197	After 24 h	ageing
30	6.26	98.9%	201	0	start

**Table 2:** Selection of TSO concentration.

Time (h)	TSO Concentration (μg/mL)								
	200	100	50	40	25	12	9	6	3
0	×	×	√	√	√	√	√	√	√
2	×	×	√	√	√	√	√	√	√
4	×	×	√	√	√	√	√	√	√
8	×	×	×	√	√	√	√	√	√
24	×	×	×	×	×	×	√	√	√
48	×	×	×	×	×	×	√	√	√

## Data Availability

All the datasets on which the conclusions of the manuscript rely are presented in the paper.

## Supplementary Materials

The following are available online at https://www.mdpi.com/article/10.3390/ijms222212428/s1.
