# Supplementary figures and images for: Tryptophan Side-Chain Oxidase Enzyme Suppresses Hepatocellular Carcinoma Growth through Degradation of Tryptophan
Source: Int J Mol Sci. 2021 Nov 18;22(22):12428. doi: 10.3390/ijms222212428 (PMC8623686; doi:10.3390/ijms222212428)

## The Supplementary figures

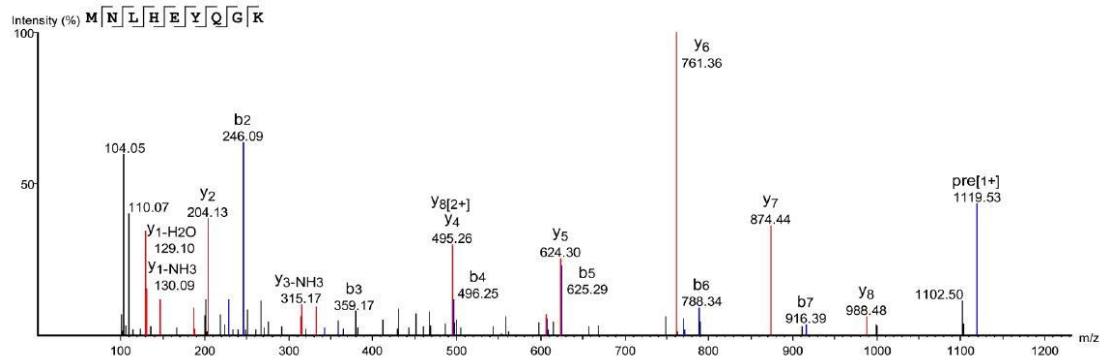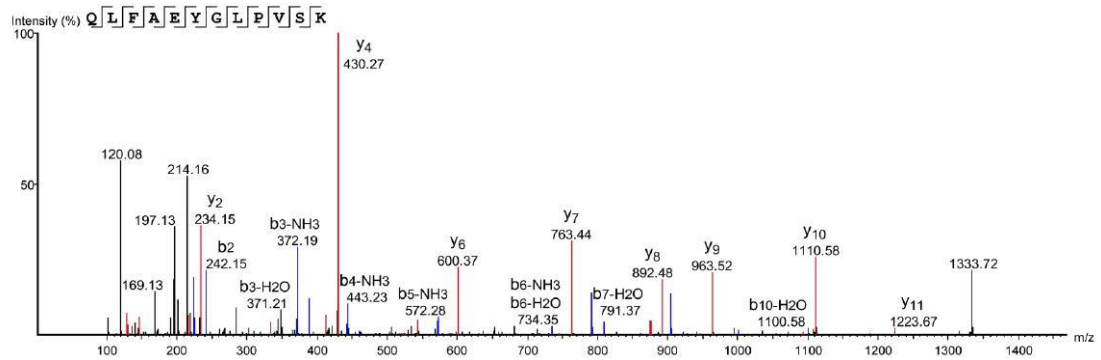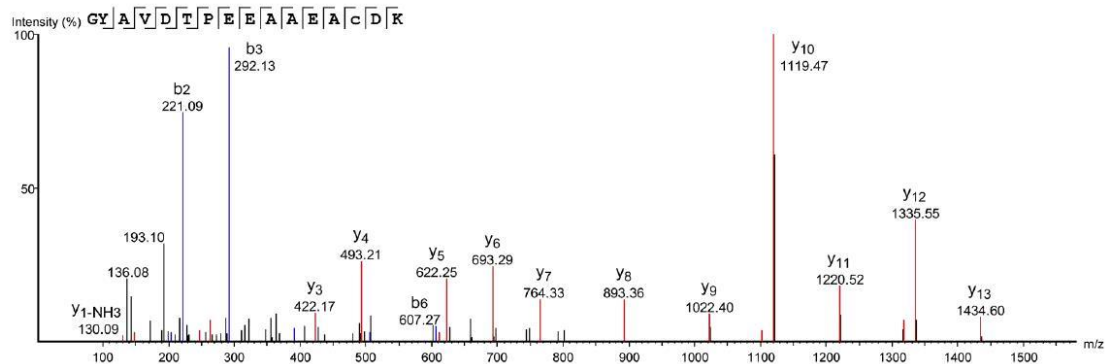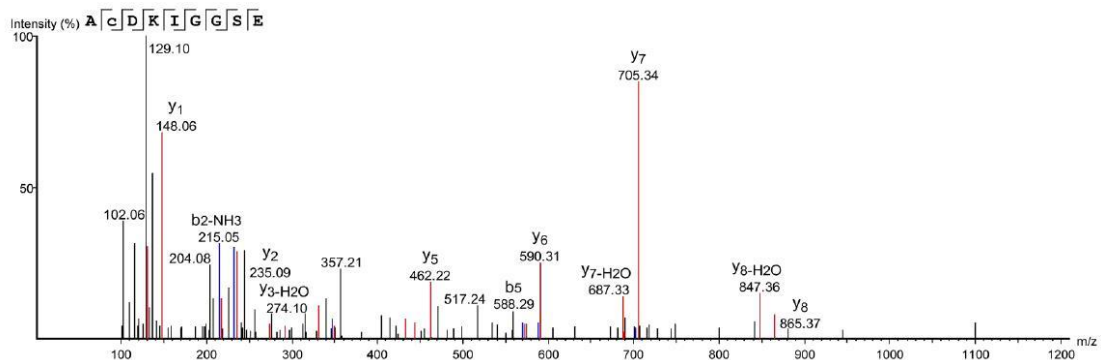

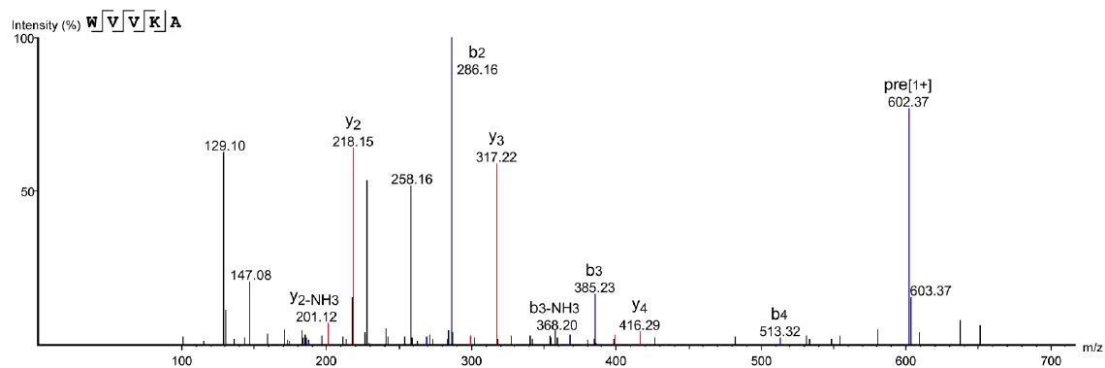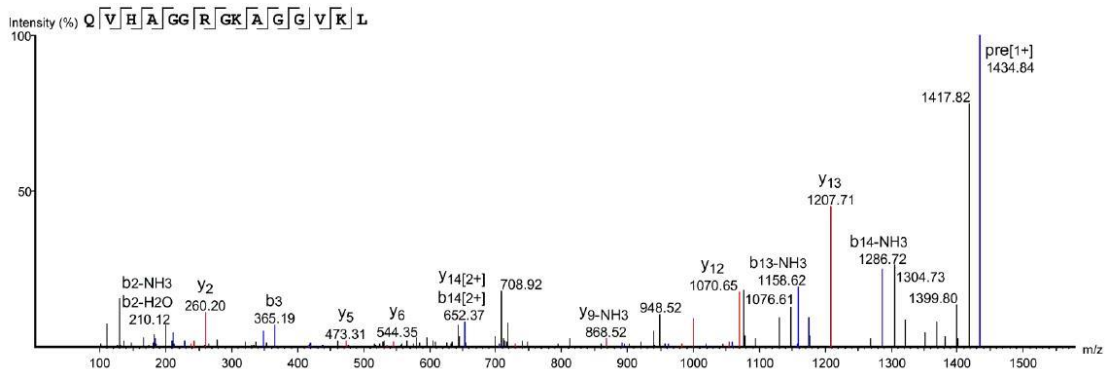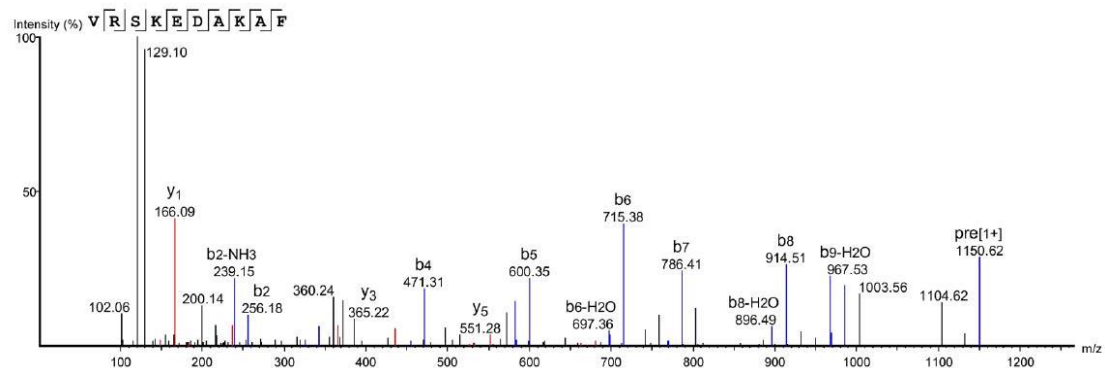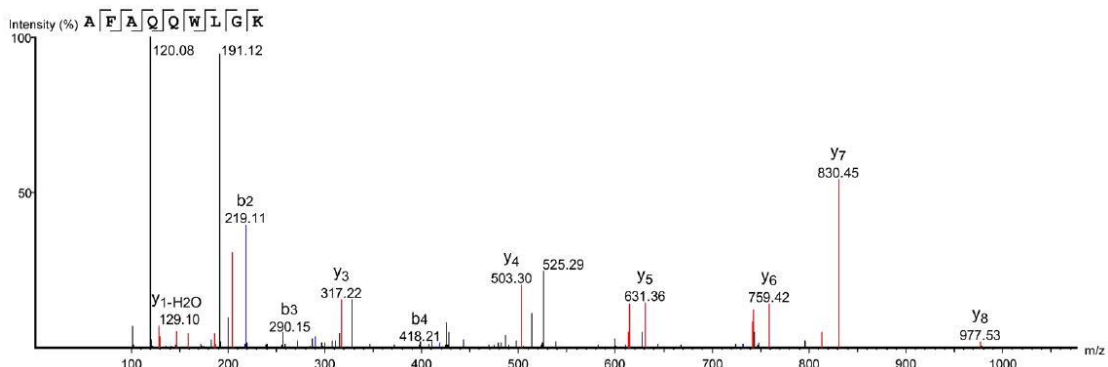

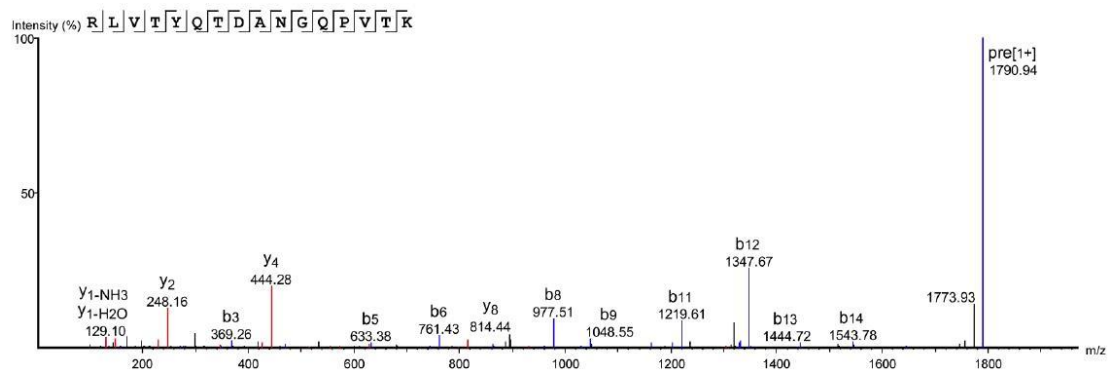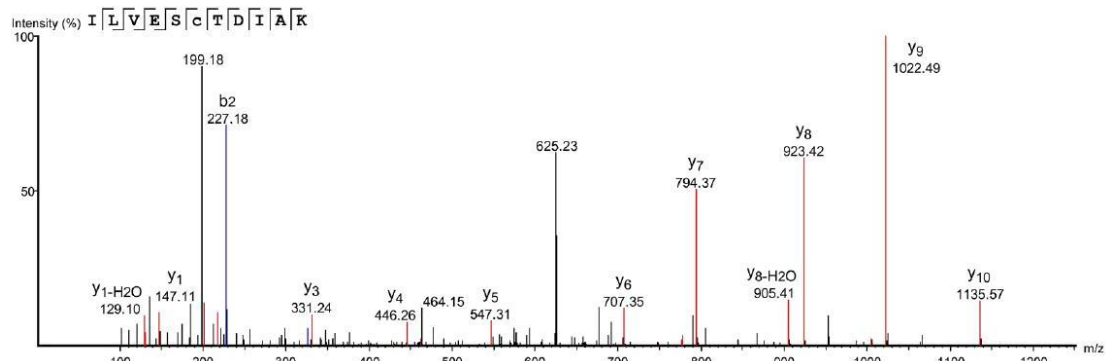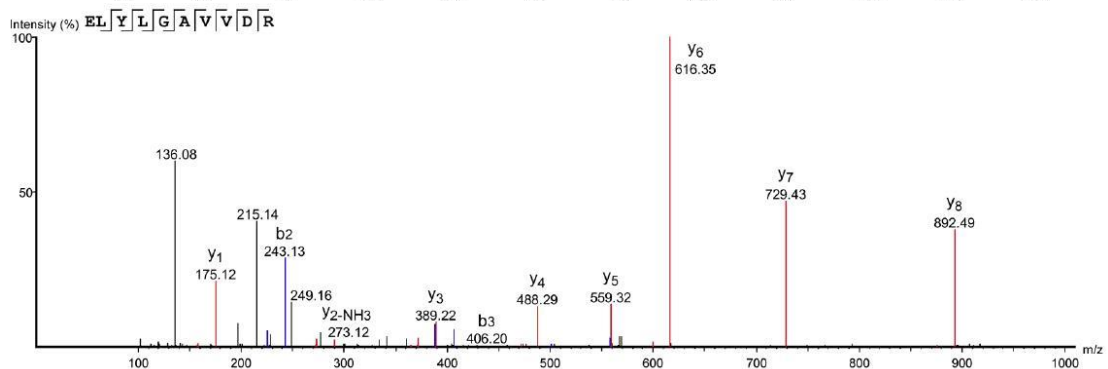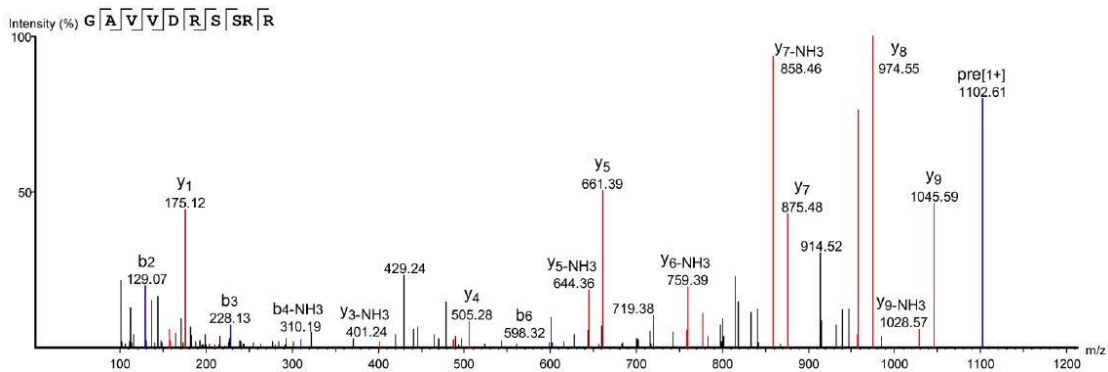

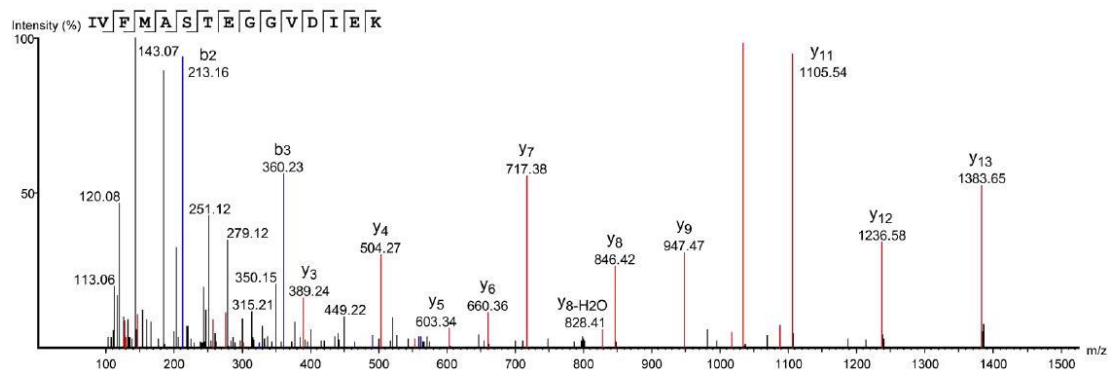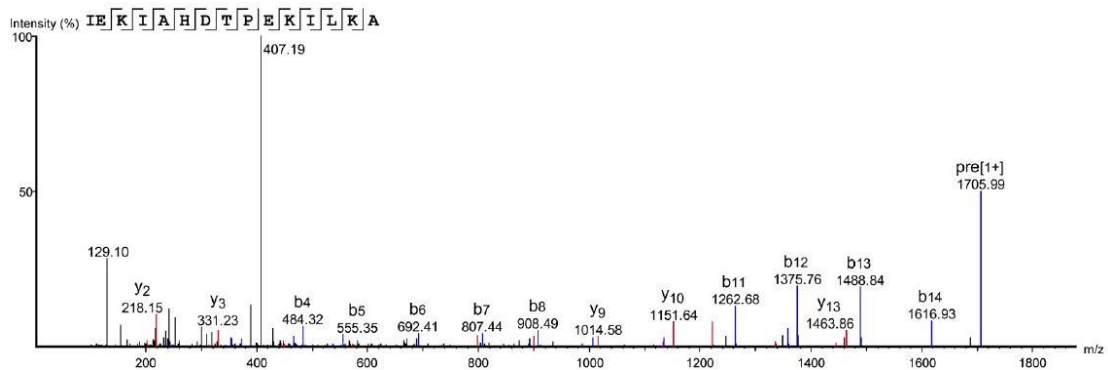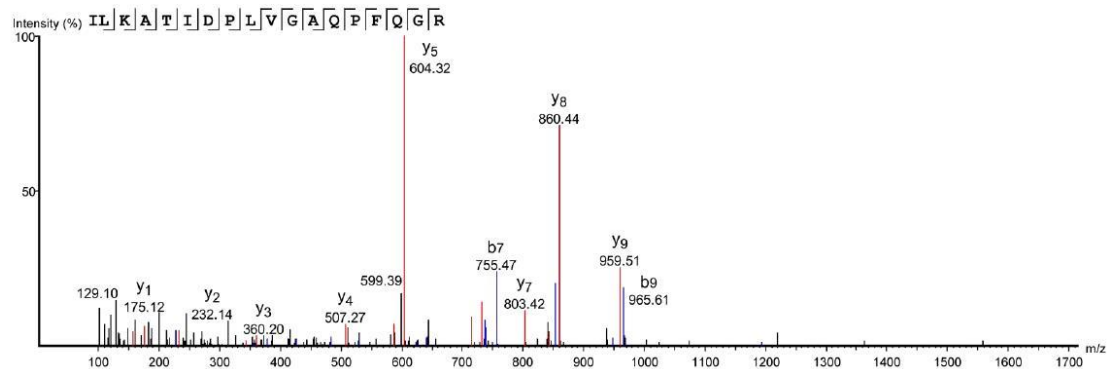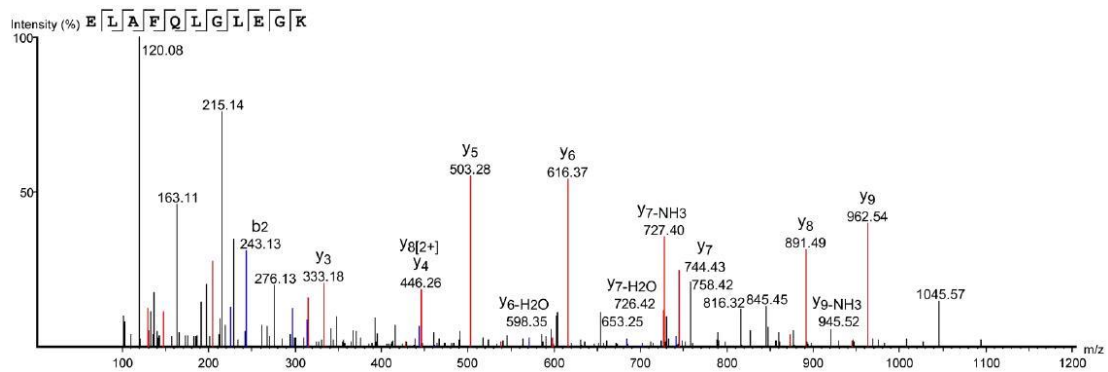

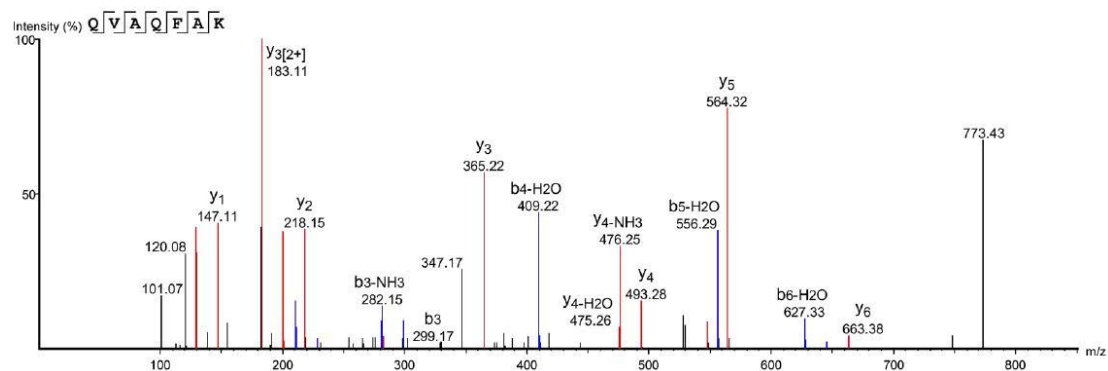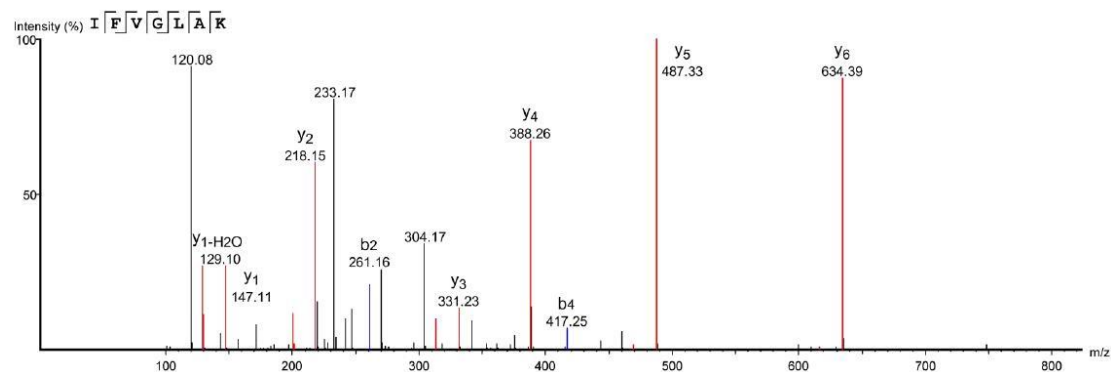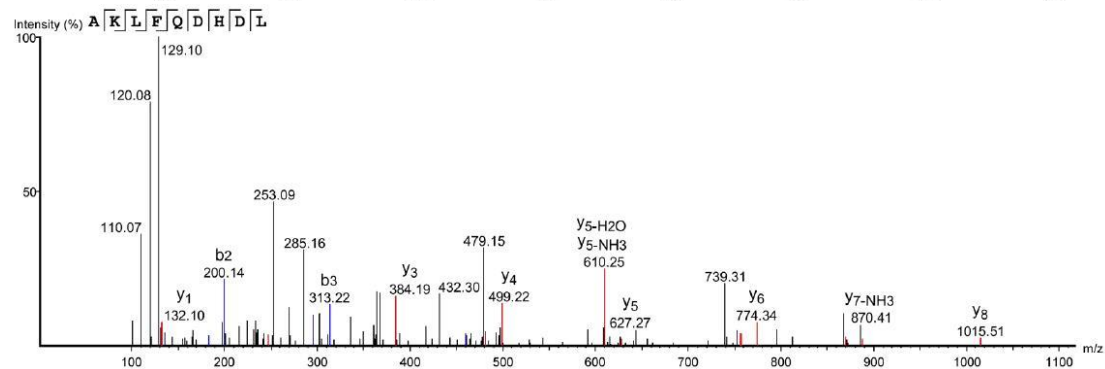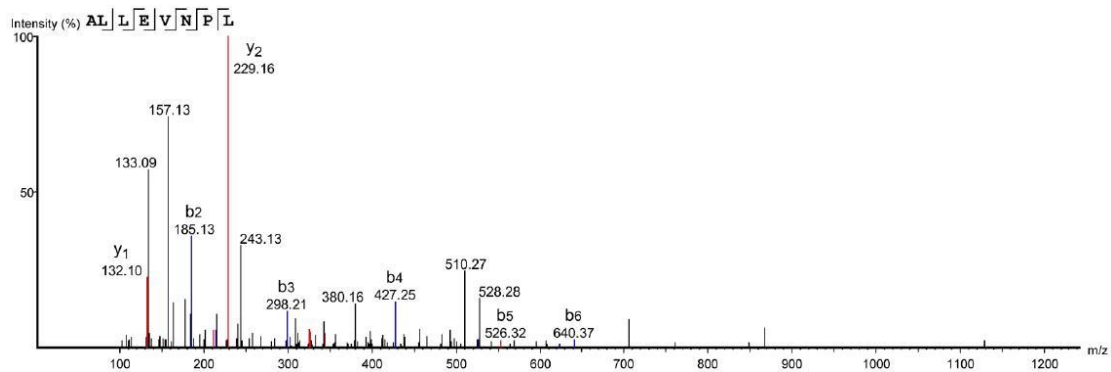

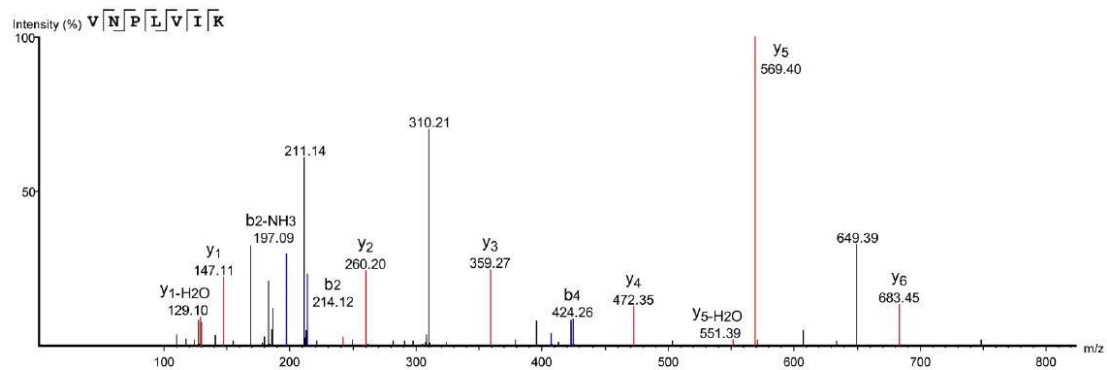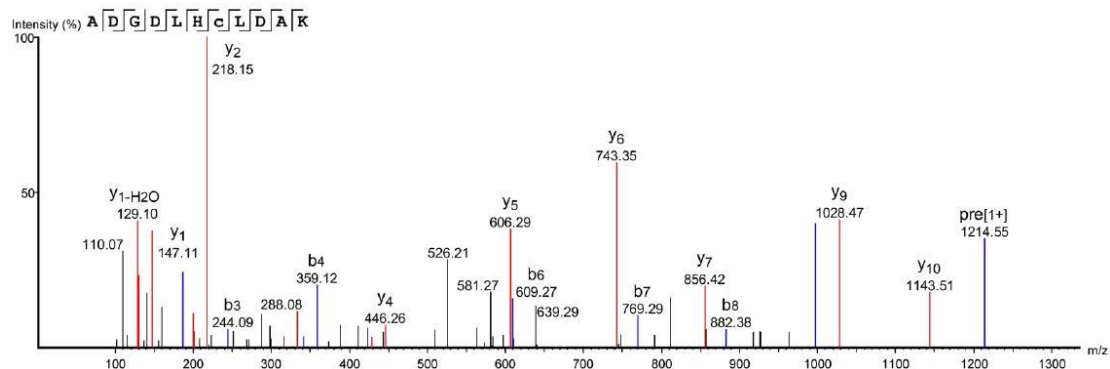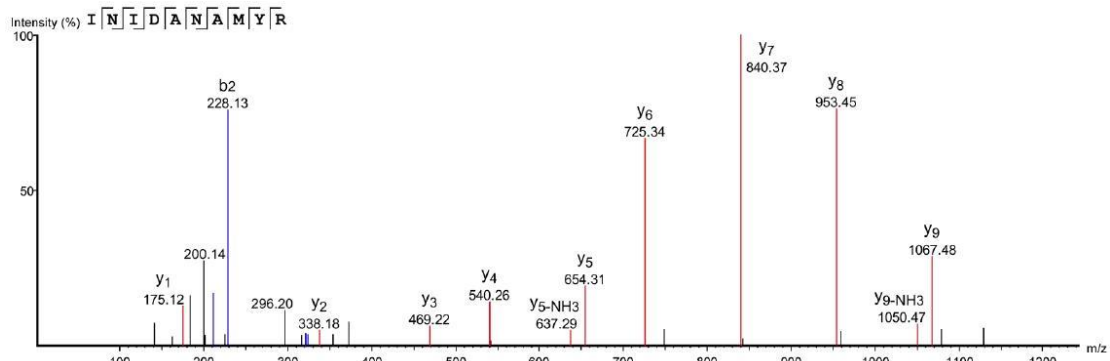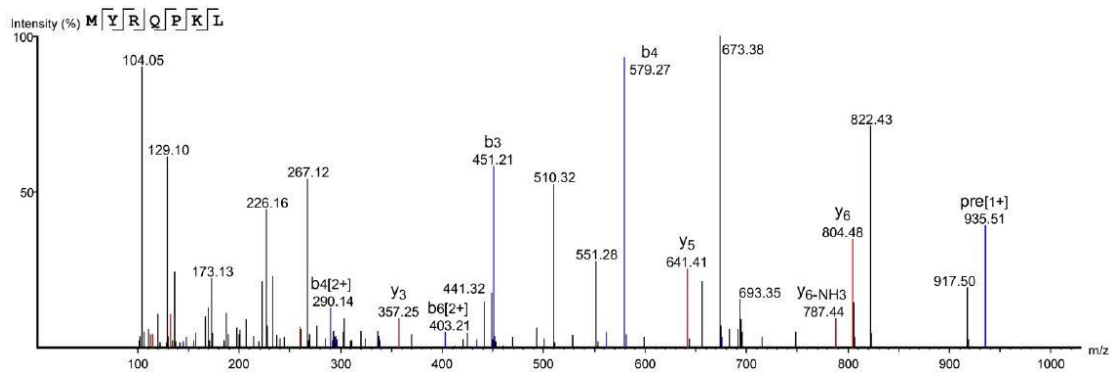

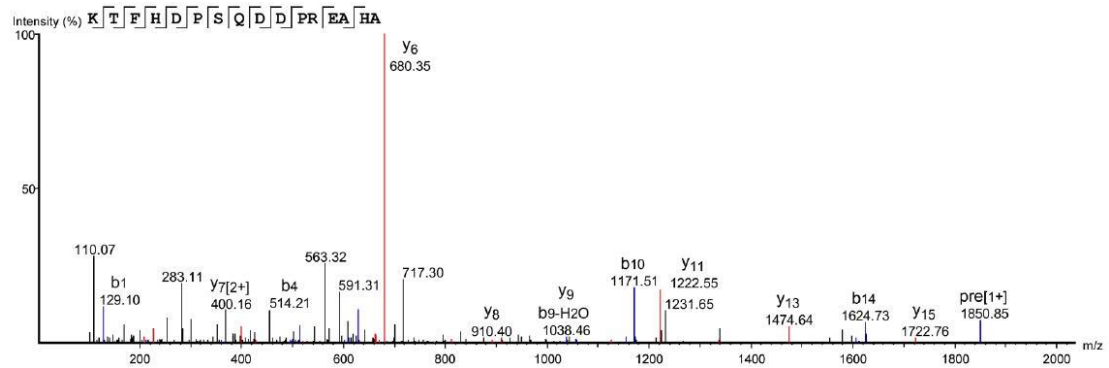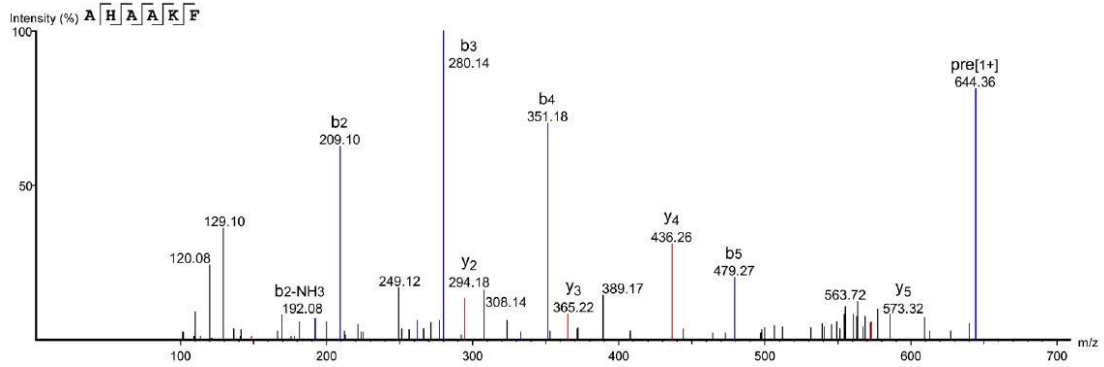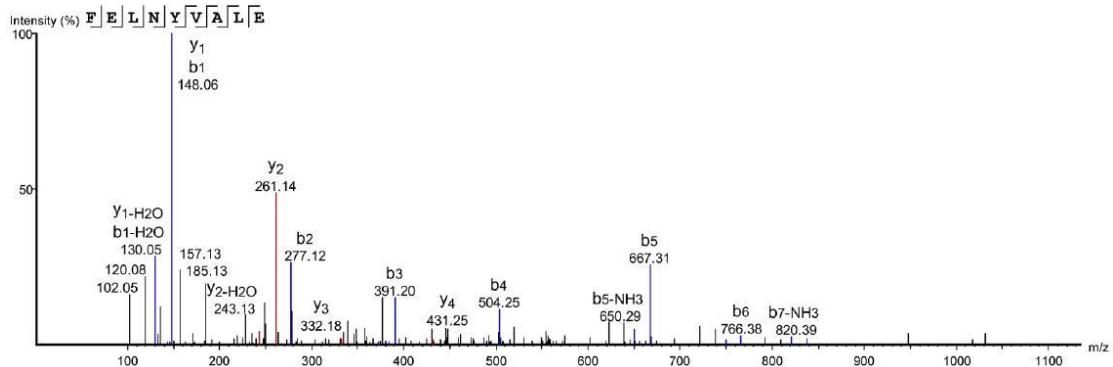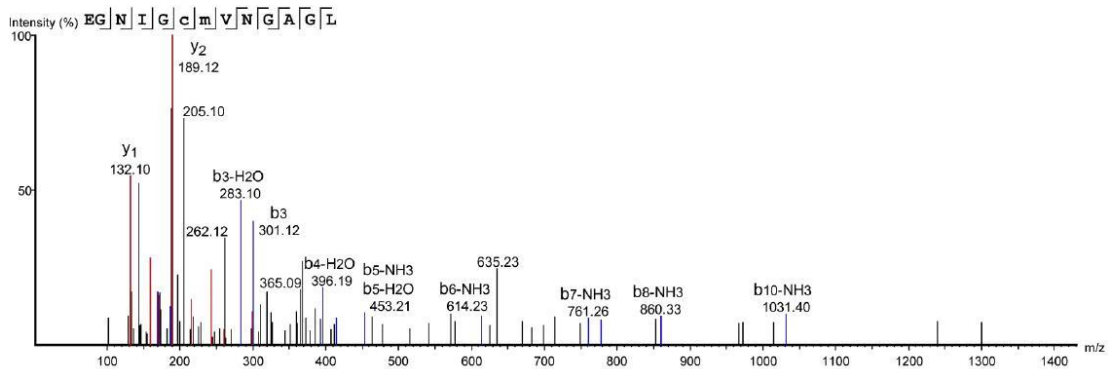

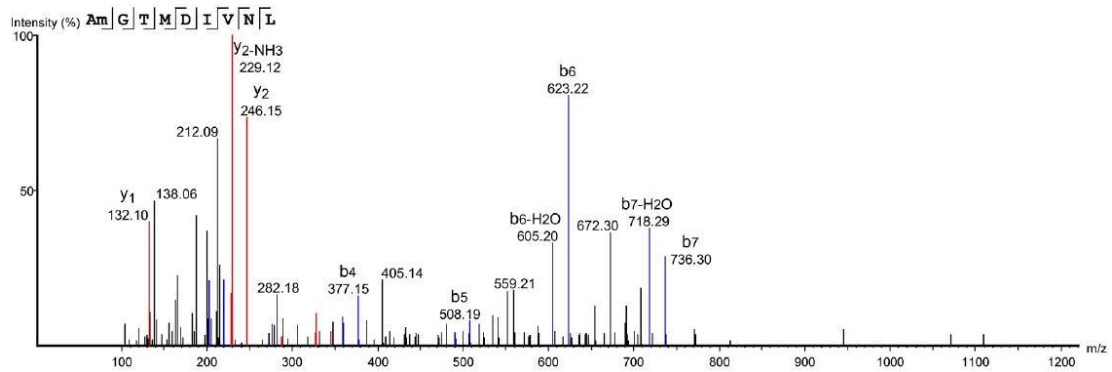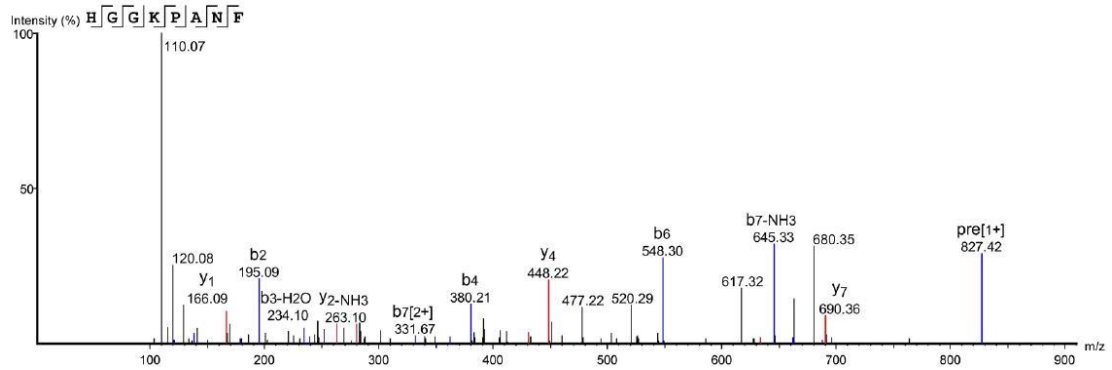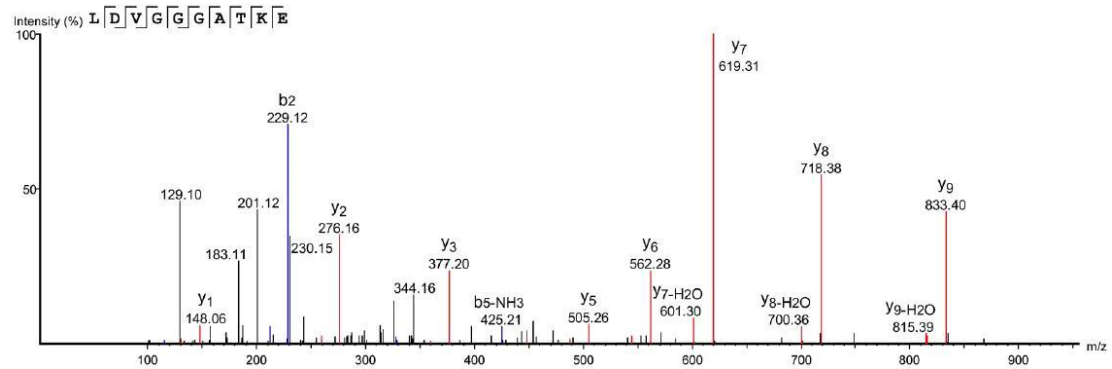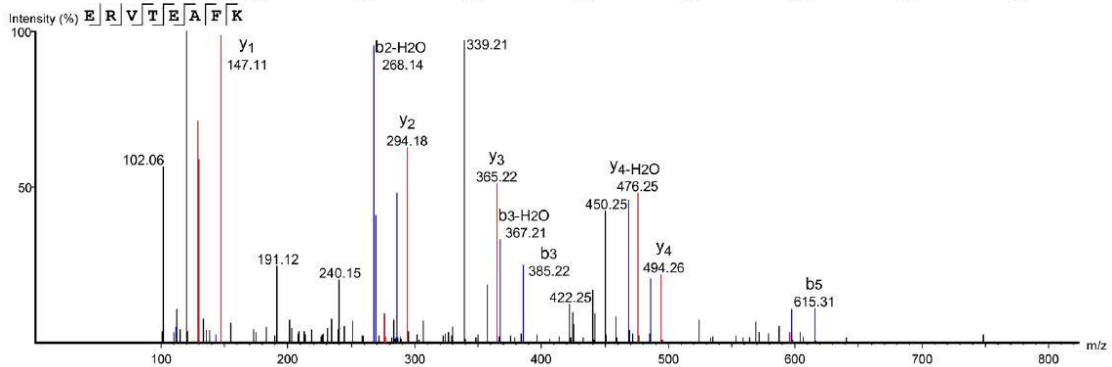

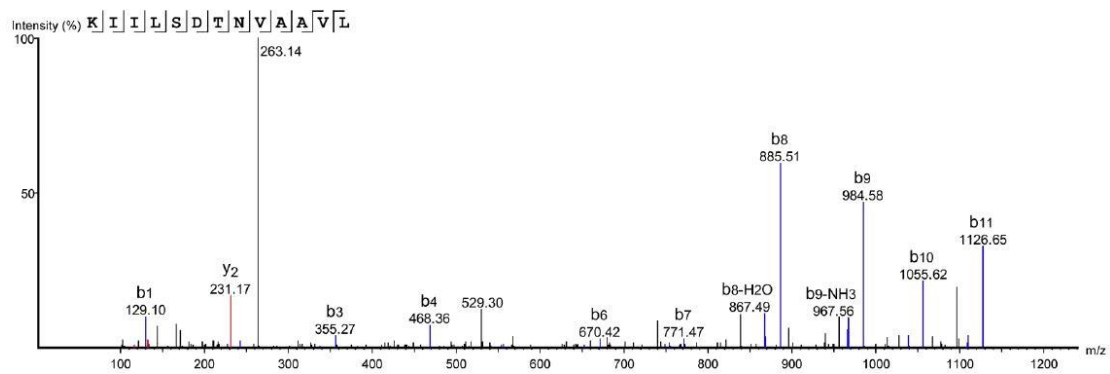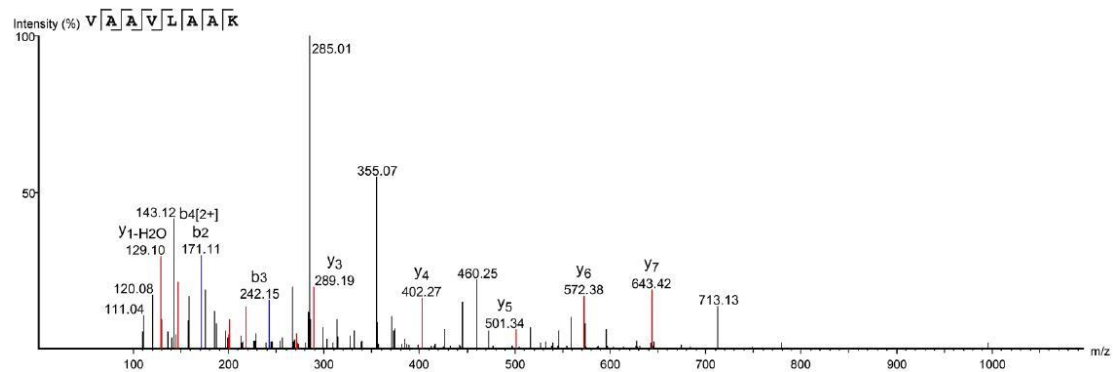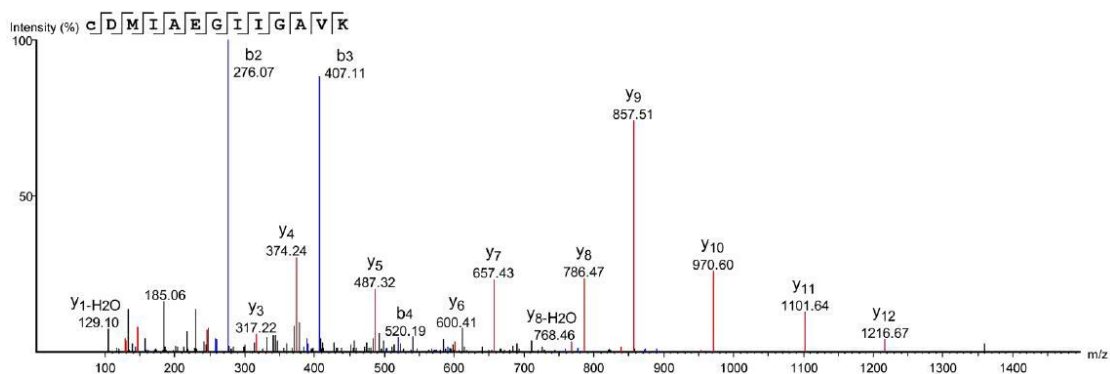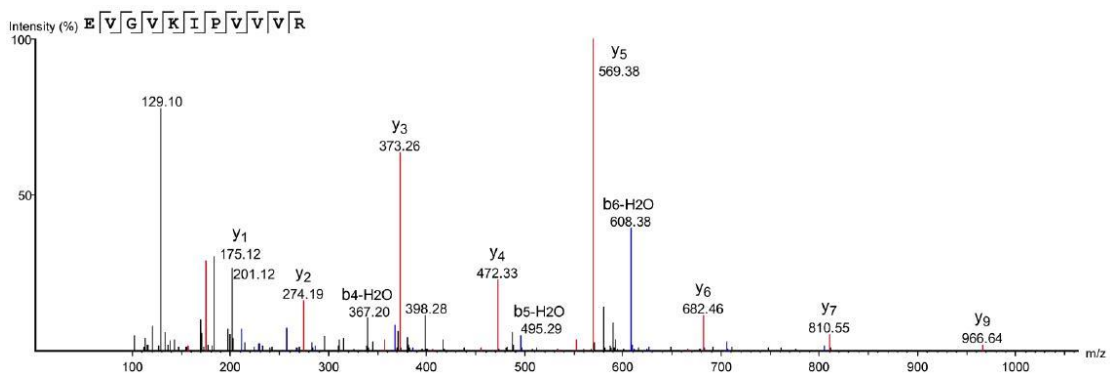

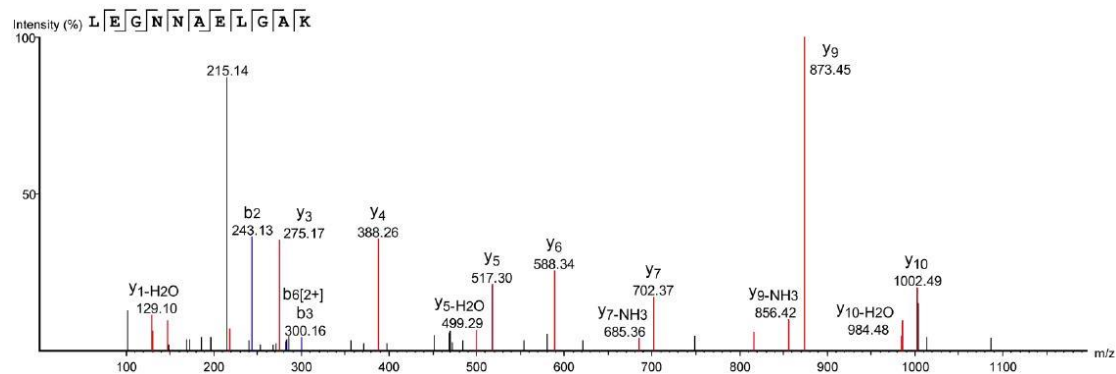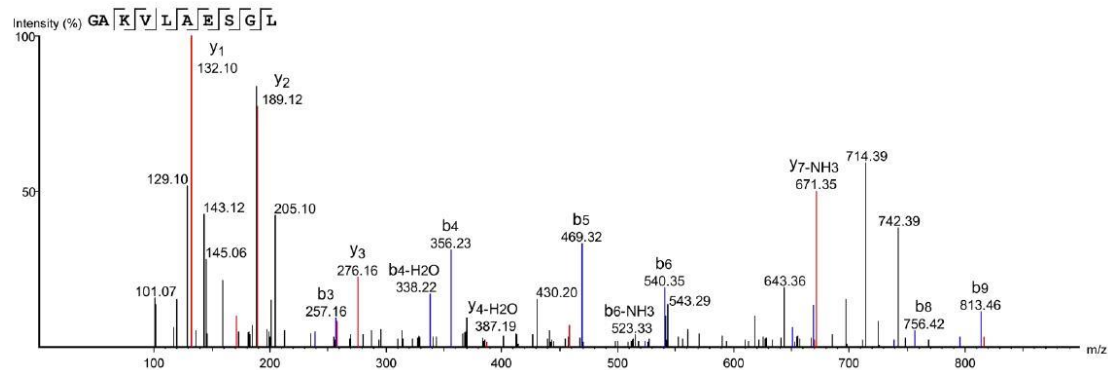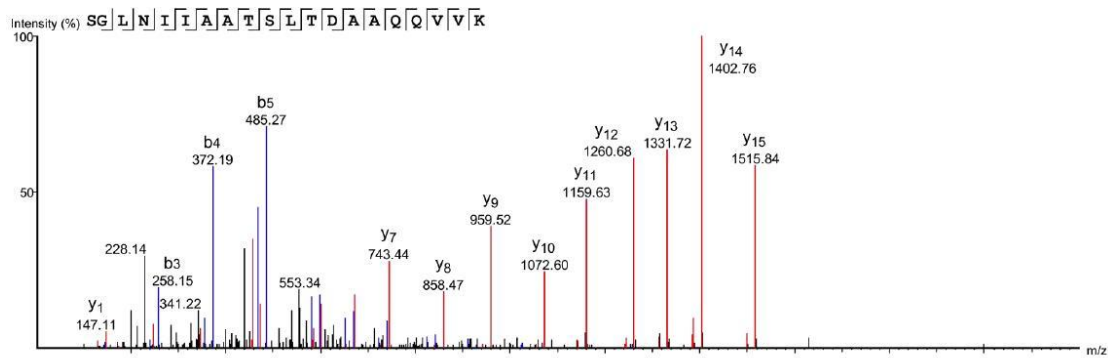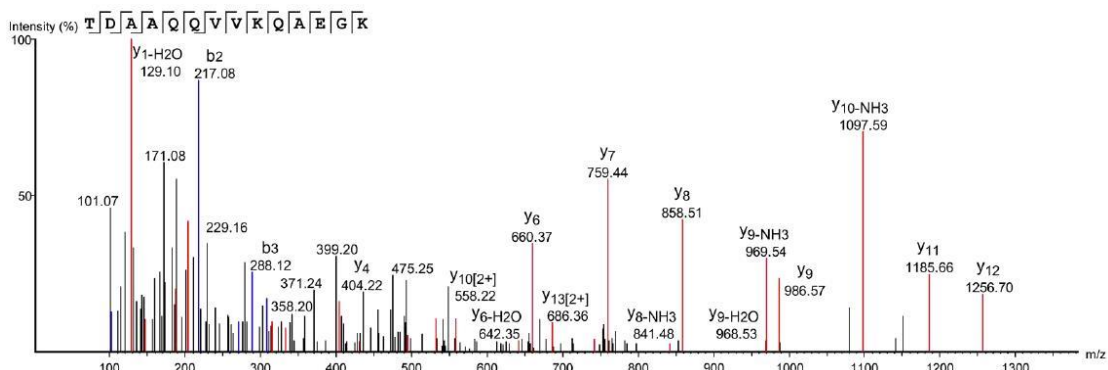

Supplement: Supplementary file 1 [file ijms-22-12428-s001.zip › ijms-1455676-supplementary.pdf]
